# Zinc pyrithione is a potent inhibitor of PL^Pro^ and cathepsin L enzymes with *ex vivo* inhibition of SARS-CoV-2 entry and replication

**DOI:** 10.1080/14756366.2022.2108417

**Published:** 2022-08-09

**Authors:** Jerneja Kladnik, Ana Dolinar, Jakob Kljun, David Perea, Judith Grau-Expósito, Meritxell Genescà, Marko Novinec, Maria J. Buzon, Iztok Turel

**Affiliations:** a Faculty of Chemistry and Chemical Technology, University of Ljubljana, Ljubljana, Slovenia; b Infectious Diseases Department, Vall d’Hebron Research Institute (VHIR), Hospital Universitari Vall d’Hebron, Universitat Autònoma de Barcelona, VHIR Task Force COVID-19, Barcelona, Spain

**Keywords:** Antiviral agents, SARS-CoV-2, inhibition, pyrithione, zinc

## Abstract

Zinc pyrithione (**1a**), together with its analogues **1b**–**h** and ruthenium pyrithione complex **2a**, were synthesised and evaluated for the stability in biologically relevant media and anti-SARS-CoV-2 activity. Zinc pyrithione revealed potent *in vitro* inhibition of cathepsin L (IC_50_=1.88 ± 0.49 µM) and PL^Pro^ (IC_50_=0.50 ± 0.07 µM), enzymes involved in SARS-CoV-2 entry and replication, respectively, as well as antiviral entry and replication properties in an *ex vivo* system derived from primary human lung tissue. Zinc complexes **1b**–**h** expressed comparable *in vitro* inhibition. On the contrary, ruthenium complex **2a** and the ligand pyrithione **a** itself expressed poor inhibition in mentioned assays, indicating the importance of the selection of metal core and structure of metal complex for antiviral activity. Safe, effective, and preferably oral at-home therapeutics for COVID-19 are needed and as such zinc pyrithione, which is also commercially available, could be considered as a potential therapeutic agent against SARS-CoV-2.

## Introduction

Research and development of safe and effective pharmacotherapy for a life-threatening COVID-19 disease must now focus on curative approaches after the rapid and successful development of SARS-CoV-2 vaccines. The development of specific SARS-CoV-2 inhibitors requires a thorough knowledge of viral structure, host cell entry, and the replication cycle^1^. SARS-CoV-2 is an enveloped single-stranded positive-sense RNA virus^2^ that requires binding of the viral spike protein to the human zinc metalloproteinase receptor ACE2 as the first step in internalisation process^3^. After binding, viral entry is possible through (i) direct fusion with the cell surface involving the proteases furin and transmembrane serine protease-2 (TMPRSS2), followed by direct release of SARS-CoV-2 RNA and/or (ii) receptor-mediated endocytosis, in which the lysosomal cysteine protease cathepsin L facilitates the release of the genetic material after fusion of the viral and endosomal membranes^4^. Once RNA is released into the cytosol, translation and replication of the genome occur, including the potential targets papain-like protease (PL^Pro^), chymotrypsin-like protease (3CL^Pro^, also abbreviated as M^Pro^), and RNA-dependent RNA polymerase (RdRp). After the synthesis of the viral structural proteins and their transition through endoplasmatic-reticulum-to-Golgi intermediate compartment, the viral copies are released from host cells by exocytosis^5^.

To our knowledge, the following intravenous/subcutaneous treatments are currently approved by EMA and FDA: remdesivir targeting RdRp, and antibodies targeting the spike protein, i.e. casirivimab/imdevimab, tixagevimab/cilgavimab, regdanvimab, bamlanivimab/etesevimab, and sotrovimab as well as tocilizumab as an interleukin-6 blocker. In addition, anakinra, a subcutaneously applied interleukin-1 receptor antagonist and orally administered PF-07321332 (SARS-CoV-2-M^Pro^ inhibitor) along with ritonavir (which slows down the metabolism of PF-07321332) were also authorised for COVID-19 treatment. Currently, molnupiravir (a ribonucleoside analogue that inhibits SARS-CoV-2 replication) and baricitinib (an immunosuppressant) are awaiting the green light for their marketing authorisation^6^^,^^7^. However, due to constant viral mutations, it is necessary to continue research and development of new multimodal therapeutic options with other targets. To skip the rigorous and lengthy process from potential drug candidate to its clinical approval, many investigations have been conducted to repurpose already approved drugs for COVID-19 indication^8^. The calpain inhibitors II and XII are such examples of potential SARS-CoV-2 therapeutics that provide dual inhibition of MPro and cathepsin L^9^.

Zinc is the second most abundant d-block metal in the human body and plays several important roles in biological systems^10^. Zinc received much attention during the pandemic because of its antiviral, anti-inflammatory, and antioxidant properties^11^. Many studies suggest that zinc deficiency increases susceptibility to infectious diseases, including COVID-19^12–15^. In addition, hypozincemia has also been suspected to cause dysgeusia and anosmia, common COVID-19 symptoms^16^^,^^17^ and also negatively affect the activity of signalling molecules leading to cytokine storm and severe lung damage^18^. There is some evidence that Zn^2+^ can indirectly inhibit ACE2 receptors^11^^,^^18^^,^^19^ and furin^20^. A computational study has confirmed possible inhibition of RdRp and M^Pro^ by zinc ions^21^^,^^22^. Additionally, the crystal structure of SARS-CoV-2 M^Pro^ has confirmed the interactions of Zn^2+^ in the active site^23^^,^^24^. Although the structural studies clearly confirmed that a zinc ion inhibits SARS-CoV-2 M^Pro^ by binding to the active site, it was also found that the experimentally determined affinity between the protein and the metal ion does not appear to be high enough for any real therapeutic applications of zinc supplementation, at least in its ionic form^23^.

**Scheme 1.**
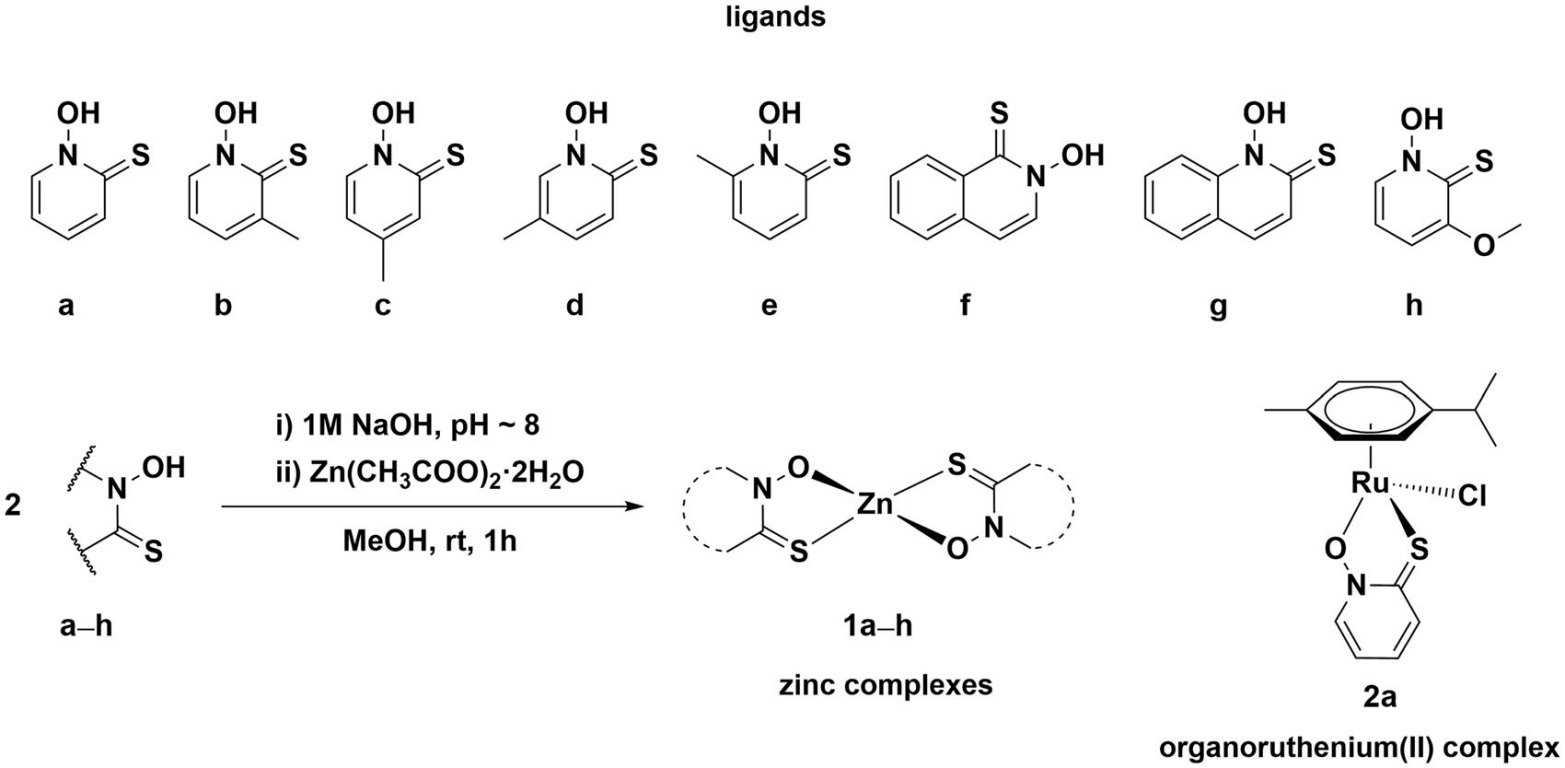
Chemical structures of investigated pyrithionato ligands **a**–**h**, synthesis of zinc complexes **1a**–**h** and organoruthenium complex **2a**.

In 2010, te Velthuis et al. showed that the combination of Zn^2+^ ions and pyrithione **a** (1-hydroxy-2(1*H*)-pyridinethione or 2-mercaptopyridine *N*-oxide, Scheme 1) successfully inhibited SARS-CoV replication, possibly by Zn^2+^ suppression of RdRp activity. Inhibition of SARS-CoV was less potent with pyrithione itself and even less effective with zinc acetate alone^25^. Zinc pyrithione **1a** has also been shown to effectively inhibit M^Pro^ and PL^Pro^ of SARS-CoV^26–28^ as well as M^Pro^ of SARS-CoV-2^29–31^ and SARS-CoV-2 PL^Pro^ (however, IC_50_ value was not determined)^31^. There are several reports that zinc pyrithione damages iron–sulphur (Fe–S) clusters and consequently elicits antimicrobial activity^32–34^. Recently, Maio et al. suggested that Fe–S clusters act as cofactors of the SARS-CoV-2 RdRp enzyme and therefore represent another potential target to combat COVID-19^35^.

Based on the described data from the literature, we decided to further investigate a system of zinc and pyrithione as a promising antiviral combination against SARS-CoV-2. Our group has previously conducted an extensive research on biological properties of organoruthenium(II) pyrithionato complexes^36–39^, however, zinc appears to be more suitable to combat COVID-19. In addition to antiviral properties, zinc pyrithione has been thoroughly investigated before for other antimicrobial properties and is now used in commercial anti-dandruff shampoos^33^, and as antifouling agent in paints^40^.

In this study, a set of zinc compounds containing the ligand pyrithione and its analogues (**1a**–**h**) was prepared and tested for the anti-SARS-CoV-2 activity. In addition, the ligands themselves (**a**–**h**) and the ruthenium complex with pyrithione **2a** were included to better understand the role of the metal centre (Scheme 1). Based on the excellent results of *in vitro* inhibition of enzymes cathepsin L and PL^Pro^, involved in SARS-CoV-2 life cycle, further *ex vivo* assays were performed to investigate the inhibition of SARS-CoV-2 entry as well as its replication.

## Methods

### Synthesis

Protocols for the synthesis of the ligands and complexes are described in the Supplementary information (SI) file.

### UV–vis stability

The same buffers as for enzymatic assays were used for UV–vis stability testing (50 mM acetate buffer, pH = 5.5, and 50 mM HEPES buffer, pH = 7.4). Pyrithione **a** and zinc pyrithione **1a** were dissolved in DMSO. Mixtures of a dissolved compound in DMSO (1%) and buffer (99%) were filtered through Minisart^®^ Regenerated Cellulose Syringe Filter (25 mm, 0.2 µm). Ruthenium complex **2a** was dissolved directly in buffers. After preparation, the spectra were immediately recorded on UV–vis spectrometer with an additional recording after 30 min.

### NMR stability

Acetate and HEPES buffers were prepared in D_2_O. Pyrithione **a** and zinc pyrithione **1a** were first dissolved in DMSO-d_6_ to which selected buffer in D_2_O was added to obtain final 1% DMSO-d_6_/acetate buffer or 1% DMSO-d_6_/HEPES buffer solutions. Such solutions were further filtered through Minisart^®^ Regenerated Cellulose Syringe Filter (25 mm, 0.2 µm). Ruthenium complex **2a** was dissolved in deuterated buffers only without DMSO-d_6_ addition and filtration. ^1^H NMR spectra were recorded immediately after solution preparations and after 30 min.

### Protein expression and isolation

Recombinant procathepsin L was prepared according to the described protocol^41^. Recombinant PL^Pro^ was prepared according to the modified protocol^42^. Briefly, the sequence was codon optimised, synthesised and cloned into pET28b/32 (pET28b with ampicillin resistance) using *Nco*I and *Xho*I sites. The resulting plasmid was transformed into *E. coli* strain BL21(DE3) and cells were cultured in LB medium with ampicillin (50 µg/ml) at 37 °C to an OD_600_ of 0.7. PL^Pro^ expression was induced by adding 0.5 mM IPTG and 1 mM ZnCl_2_ and cells were further grown overnight with shaking at 18 °C. They were collected by centrifugation for 10 min at 8000×*g* and the pellet was resuspended in 50 mM Tris–HCl, 150 mM NaCl, 10 mM imidazole, and pH 8.5. Afterwards, the cells were lysed by homogenisation and sonication and the lysate was clarified by centrifugation at 30,000×*g* for 20 min. Supernatant was filtrated and then loaded onto a Ni-affinity column, pre-equilibrated with lysis buffer. Bound proteins were eluted in a linear imidazole gradient (10–250 mM) using chromatographic system. Fractions containing PL^Pro^ were collected and dialysed against 20 mM Tris, 100 mM NaCl, 2 mM DTT, and pH 8.5. The protein suspension was aliquoted and stored at −80 °C.

### Enzyme assays

Autocatalytical activation of procathepsin L was performed at pH ∼4. To 1 volume (V) of procathepsin, 5% V of 3 M acetate buffer, pH 3.8 was added and the mixture was then incubated at 37 °C. Enzyme activity was monitored using fluorogenic substrate. When maximum activity was reached, the activation was stopped by adding 10% V of a neutralising buffer, 3 M acetate buffer, pH 5.5.

Reaction mixture for cathepsin L constituted of 50 mM acetate buffer, pH 5.5, 2 µM Z-LR-AMC, 5 µM DTT, and compound dissolved in DMSO (final concentration 0–100 µM, up to 1% DMSO). PL^Pro^ reaction mixture was 50 mM HEPES buffer, pH 7.4, 50 µM Z-RLRGG-AMC, 5 µM DTT, and compound dissolved in DMSO (final concentration 0–100 µM, up to 1% DMSO). Positive controls for enzyme assays were reaction mixtures with 1% DMSO without the tested compound.

All enzymatic assays were performed at 25 °C with constant magnetic stirring. Hydrolysis of fluorogenic substrates was monitored at *λ*_ex_ 370 nm and *λ*_em_ 455 nm. Data were analysed and visualised in GraphPad Prism 9 (La Jolla, CA). Reaction rate *v_z_* was defined using the following equation: $Y=\;v_{s}*X+(v_{z}-v_{s})*\frac{1-e^{-k*X}}{k}+d,$ where *Y* represents measured fluorescence and *X* represents time points. Obtained reaction rates were corrected for the inner filter effect (IFE) according to the published procedure^43^. IC_50_ values were calculated using the following equation: $Y=v_{0}-\frac{(v_{0}-v_{\infty })*X}{{\mathrm{IC}}_{50}+X},$ where *Y* represents IFE corrected reaction rate and *X* represents concentration of a complex. Relative reaction rates for the ligands were determined as ratio of $v_{100\;\mu M\;\mathrm{ligand}}$ over $v_{0}.$

### Cell-based assays

#### Entry viral assay

Triplicates of the dilutions 30, 10, 1, and 0.5 μM of the antiviral compounds **a**, **1a**, and **2a** were tested for their antiviral effect in human lung tissue (HLT) cells using three different donors. Pyrithione **a** was also tested in combination with zinc acetate dihydrate (ZnAc) at different concentrations (2, 20, and 60 μM of pyrithione **a**), but maintaining a ratio of 2:1. Drug dilutions were freshly prepared in R10 in 96-well conic-bottom plates. HLT cells were obtained and processed as previously described^44^, added at a density of 300,000 cells/well and incubated with the compounds for 1 h before infection. Then, multiplicity of infection (MOI) 0.1 of VSV*ΔG(Luc)-S virus harbouring the spike from the D614G variant was added to the individual wells, and plates were spinoculated at 1200×*g* and 37 °C for 2 h. After infection, fresh medium containing the studied compounds was added to the wells, and cell suspensions were transferred into a new 96-well flat-bottom plate. Cells were then cultured overnight at 37 °C in a 5% CO_2_ incubator. Each plate contained the following controls: no cells (background control), cells treated with medium (mock infection), cells infected but untreated (infection control), and cells infected and treated with 30 μM of camostat (positive drug control). In parallel, duplicates of the tested dilutions in each assay were run to monitor drug cytotoxicity by luminescence, as previously described^44^. After 20 h, cells were incubated with Britelite plus reagent (Britelite plus kit; PerkinElmer, Waltham, MA) and then transferred to an opaque black plate. Luminescence was immediately recorded by a luminescence plate reader (LUMIstar Omega). To evaluate cytotoxicity, we used the CellTiter-Glo Luminescent kit (Promega, Madison, WI), following the manufacturer’s instructions. Data were normalised to the mock-infected control, after which IC_50_ and CC_50_ values were calculated using Graph-Pad Prism 7 (La Jolla, CA).

#### Drug validation with replication-competent SARS-CoV-2

These experiments were performed in BSL3 facilities (Centre de Medicina Comparativa i Bioimatge de Catalunya (CMCiB), Badalona, Spain). HLT tissue blocks were cultured at a density of 25–35 blocks/well in a 24-well plate and incubated with compounds **a**, **1a**, and **2a** at 30 μM for at least 1 h before infection. Then, tissue blocks were infected with a MOI 0.5 of the SARS-CoV-2 viral isolate, and the plate was incubated for 3 h at 37 °C and 5% CO_2_. After infection, samples were extensively washed with PBS 1X to eliminate residual virus and transferred into a new 24-well plate with fresh media containing the antiviral drugs. 24 h post infection, 100 μl of supernatant was collected in tubes containing 100 μl of DNA/RNA Shield (ZymoResearch, Irvine, CA) for SARS-CoV-2 inactivation. For each experiment, a negative control, cells treated with only medium, and a positive control, cells incubated in the presence of the virus alone, were included. Percentage of viral infection was calculated by RT-PCR, as previously described^44^.

A non-linear regression fit and a dose–response curve with variable slope (four parameters) were used as a statistical method in *ex vivo* assays.

### Declaration of approval for tissue experiments

Lung tissues were obtained from patients without previous COVID-19 history and a recent negative PCR test for SARS-CoV-2 infection undergoing thoracic surgical resection at the Thoracic Surgery Service of the Vall d’Hebron University Hospital. Study protocol was approved by the Ethical Committee of the Vall d’Hebron Hospital (Institutional Review Board number PR(AG)212/2020), and informed consent was obtained from all participants.

## Results

### Synthesis of pyrithiones and their zinc complexes

First, pyrithionato ligands were prepared in a two-step synthesis and then complexed with the zinc ion (Scheme 1). We prepared pyrithiones **b**–**e** with methyl substituents at different positions of the pyrithione backbone together with two pyrithiones **f**–**g** with extended aromaticity. According to the concept of hard and soft acids and bases (HSAB), zinc generally has a borderline character. It tends to form complexes with both hard oxygen and soft sulphur donors. Pyrithiones have the ability to coordinate well to various metal ions via their sulphur and oxygen donor atoms to form metal complexes, and particularly stable complexes are formed with zinc (apparent *K*_d_=5.0 × 10^−12^ M^2^).^45^

Zinc complexes **1a**–**h** were prepared by a modified published procedure^46^. First, the corresponding pyrithione ligand **a**–**h** was dissolved in methanol. Before the addition of zinc acetate, the pyrithione ligand had to be deprotonated, which was achieved with an aqueous solution of 1 M NaOH. After addition of zinc acetate to this solution, a white precipitate was obtained immediately, which was then filtered off and dried overnight in an oven. All compounds prepared were physico-chemically characterised by NMR (Supplementary Figures 3–10), high resolution mass spectrometry, elemental analysis (C, H, N), IR spectroscopy, and new crystal structures were also obtained for compounds **1d** and **1f** (see below).

### Crystal structures of zinc complexes

**Figure 1.**
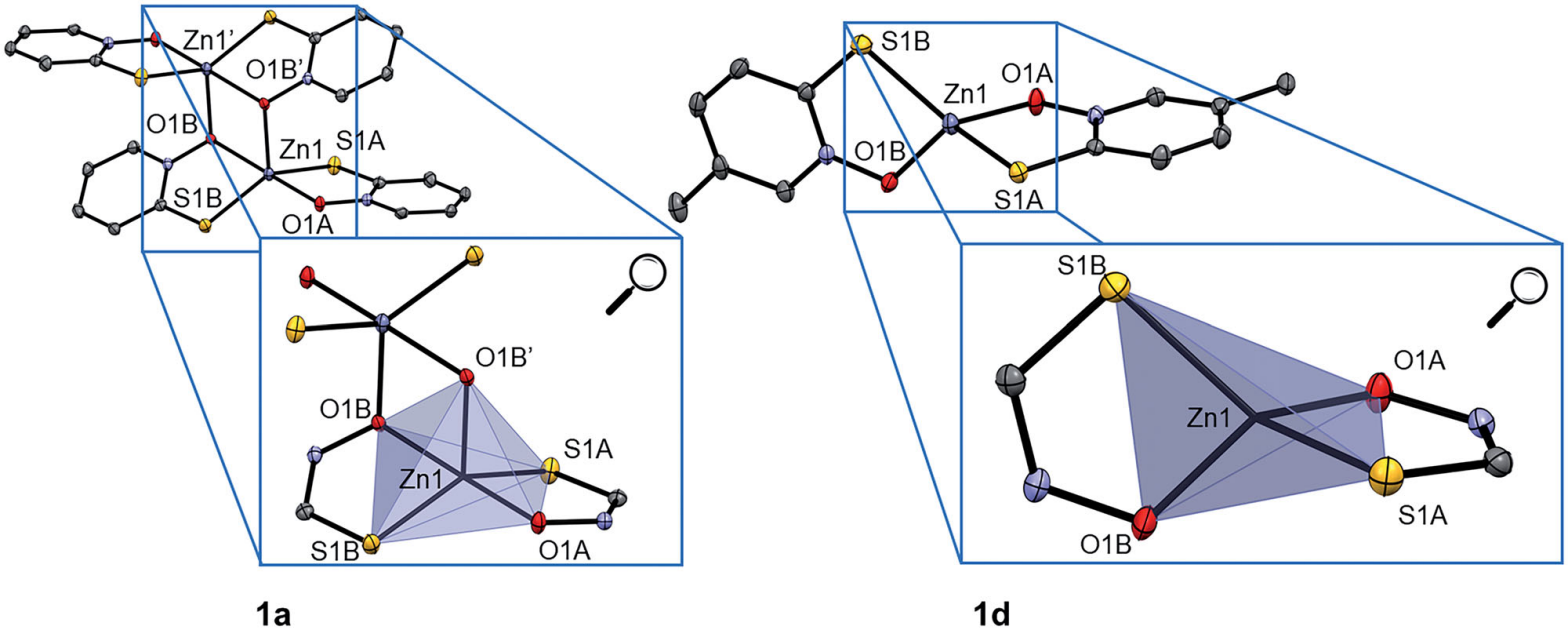
Crystal structure of complexes **1a** and **1d** (together with zoom of trigonal bipyramidal and tetrahedral coordination, respectively). Ellipsoids are drawn at the 35% probability level. Additional data can be found in SI (Supplementary Figure 2).

In the literature, it is described that the zinc pyrithione complex **1a** crystallises as a dimer with highly distorted trigonal bipyramidal geometry, where the zinc ion is coordinated by five donor atoms (Figure 1). Two pyrithionato ligands bind to the central zinc ion via *S*- and *O*-atoms to form a monomeric subunit, which is further linked by an additional Zn–O coordination bond to the adjacent subunit to form a dimer. However, in solution zinc pyrithione exists as a monomer^47^.

The crystal structure of zinc complex **1b** is comparable to that of the parent zinc pyrithione complex **1a**^48^. For complex **1c**, the crystal structure was previously published as an ethanol solvate monomer with a distorted tetrahedral geometry (Supplementary Figure 1)^49^. Complexes **1d** and **1f** crystallise as a monomers with a distorted tetrahedral geometry similar to that of previously reported complex **1e** (Figure 1, Supplementary Figures 1 and 2, Supplementary Table 2)^50^. Crystals for both complexes were obtained by liquid diffusion at room temperature using chloroform/heptane for **1d** and chloroform/dichloromethane solvent system for **1f**. Crystallographic data for new crystal structures of **1c**, **1d**, and **1f** are given in Supplementary Table 1 and an overview of selected bond lengths, angles, and calculated geometry indices (*τ*_4_, *τ*_4_′, *τ*_5_) as well as references to previously published structures is given in Supplementary Table 2. The complexes crystallise either as monomers or as dimers. The geometry in monomeric forms is distorted tetrahedral (*τ*_4_=0.70–0.78) and in dimeric forms distorted trigonal bipyramidal (*τ*_5_=0.51–0.59) (Supplementary Table 2).

### UV–vis and NMR stability in biologically relevant media

UV–vis spectroscopy was first used to determine if the compounds are stable in aqueous media and therefore suitable for further biological studies (Supplementary Figures 11–16). The same media were used as for cathepsin L and PL^Pro^ enzymatic assays, i.e. either 1% DMSO/acetate buffer (for the cathepsin L assay) or 1% DMSO/HEPES buffer (for the PL^Pro^ assay). From the UV–vis spectra, it is evident that ligand **a** and its zinc complex **1a** remain stable in acetate buffer (Supplementary Figures 11 and 12). Ruthenium complex **2a** also proved to be a stable compound in this media as well (Supplementary Figure 13). UV–vis stability measurement of the above-mentioned compounds in HEPES buffer also gave comparable curves (Supplementary Figures 14–16). Therefore, the UV–vis stability analysis provided us with encouraging results for further biological assays.

The selected compounds (**a**, **1a**, and **2a**) were further examined for their stability by ^1^H NMR spectroscopy at two selected time points appropriate for the time frame of the enzymatic assays (*t* = 0 and 30 min; Supplementary Figures 17–20). The media for compounds **a** and **1a** were either (i) 1% DMSO-d_6_/acetate buffer prepared in D_2_O (as for cathepsin L assay) or (ii) 1% DMSO-d_6_/HEPES buffer prepared in D_2_O (as for PL^pro^ assay). For ruthenium complex **2a**, both media were used without DMSO-d_6_. Ligand **a** itself is a stable organic compound, showing no structural changes when tracking stability in both media over the selected time points (Supplementary Figures 17a–b, 19a–b). Moreover, complex **1a** also proved to be stable in the media studied (Supplementary Figures 17c–d, 19c–d). Also, ruthenium complex **2a** remains stable throughout the NMR measurements (Supplementary Figures 18 and 20). These results further supported the experimental UV–vis data and confirmed that our compounds are suitable candidates for enzymatic assays.

### Enzymatic assays

**Figure 2.**
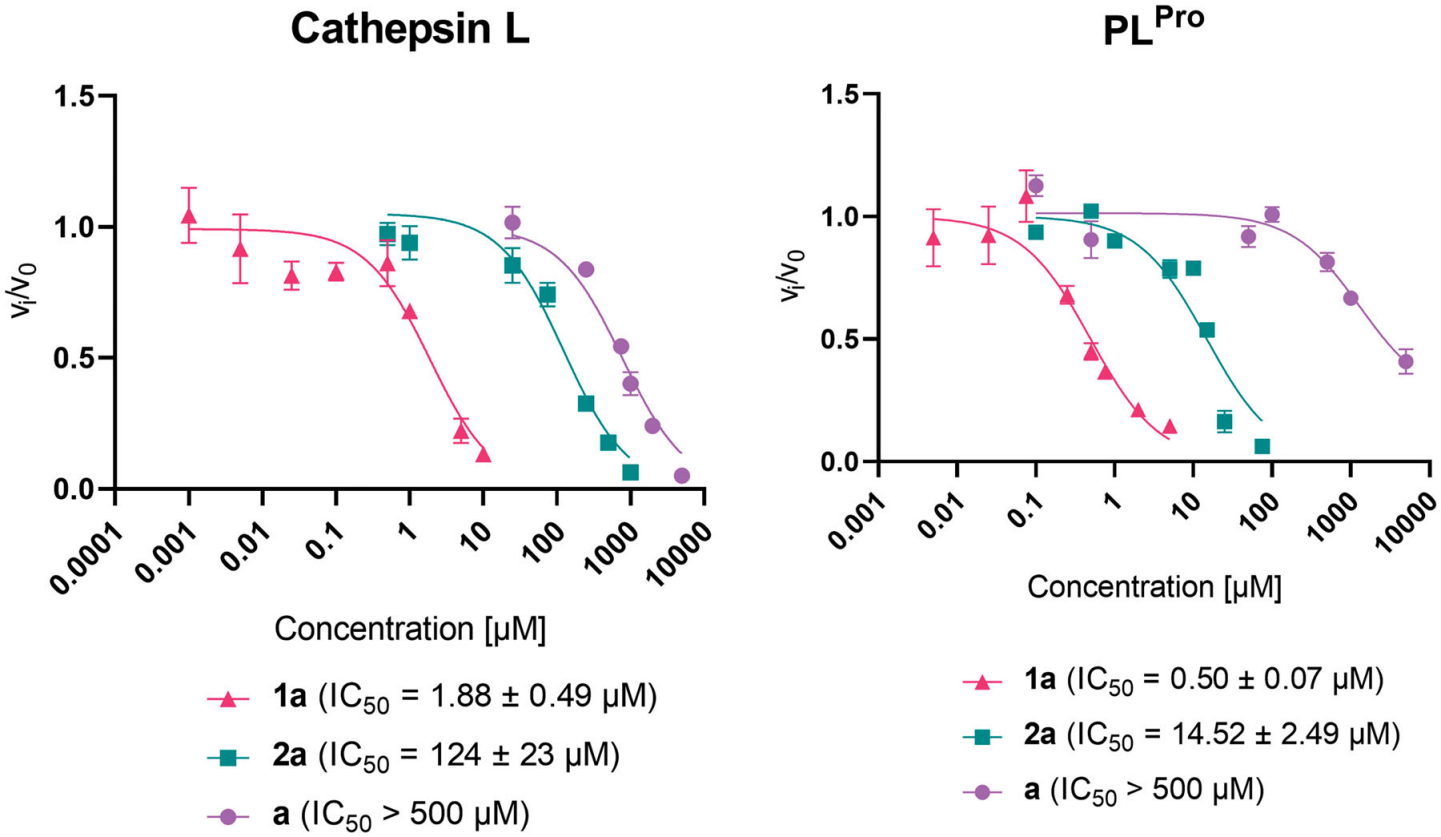
Effect of pyrithione complex with zinc **1a** and with ruthenium **2a** on cathepsin L and SARS-CoV-2 PL^Pro^. The effect of pyrithione (ligand **a**) is also shown. Data are presented as mean ± S.E.M. (*N* = 3).

We investigated the inhibitory properties of the synthesised complexes towards two enzymes involved in the development of COVID-19, namely cathepsin L and SARS-CoV-2 PL^Pro^ (Figure 2, Supplementary Figures 21 and 22, Supplementary Table 4). First, zinc complex **1a** and ruthenium complex **2a**, both of which have a pyrithione ligand bound to a metal centre, were tested to determine which metal core would be more likely to contribute to SARS-CoV-2-related target inhibition. Zinc complex **1a** inhibited both enzymes tested in the low (sub)micromolar range with IC_50_ values of 1.88 ± 0.49 µM and 0.50 ± 0.07 µM for cathepsin L and PL^Pro^, respectively. In contrast, ruthenium complex **2a** with the same pyrithione ligand showed less effective inhibition in the micromolar range with IC_50_ values of 124 ± 23 µM for cathepsin L and 14.52 ± 2.49 µM for PL^Pro^. Therefore, we decided to further investigate the inhibitory potential of analogues of zinc complex **1a**, i.e. zinc complexes with methyl-substituted pyrithiones at different positions **1b**–**e** and zinc complex with methoxy-substituted pyrithione **1h** as well as zinc complexes with pyrithiones with an extended aromatic backbone **1f**–**g**.

The introduction of a methyl group at positions 3 (**1b**), 4 (**1c**), 5 (**1d**), and 6 (**1e**) resulted in somehow stronger inhibition of cathepsin L, compared with the unsubstituted complex **1a** (IC_50_=1.88 ± 0.49 µM), with an IC_50_ of 0.35 ± 0.12 µM for **1b**, 0.41 ± 0.08 µM for **1c**, 0.44 ± 0.12 µM for **1d**, and 0.24 ± 0.05 µM for **1e**. Similar values were obtained for the complex with isoquinoline ligand (**1f**, IC_50_=0.14 ± 0.05 µM) and the complex with methoxy-substituted ligand (**1h**, IC_50_=0.74 ± 0.13 µM). Interestingly, the zinc complex with quinoline-derived pyrithione **1g** showed complex behaviour with cathepsin L that prevented IC_50_ calculation. In Supplementary Figure 21, it can be observed that complex **1g** has a dual mode of action. At concentrations below 0.1 µM and above 1 µM it acts as inhibitor, whereas in the intermediate concentration range it acts as activator. In addition, it is important to note that the ligands themselves are much weaker cathepsin L inhibitors compared to their zinc complexes (Supplementary Table 3).

A reverse trend was observed for PL^Pro^ activity in the case of zinc complex **1b** with methyl-substituted pyrithione at position 3, which slightly decreased the inhibitory activity of complex **1b** (IC_50_=1.09 ± 0.62 µM) compared to **1a** (IC_50_=0.50 ± 0.07 µM). On the other hand, the methyl substituents at the positions 5 and 6 contributes to increased inhibitory strength of complex **1d** and **1e** (IC_50_=0.22 ± 0.08 µM and 0.26 ± 0.18 µM, respectively), similar to complex **1f** with isoquinoline substituent (IC_50_=0.26 ± 0.10 µM) and complex **1h** with methoxy substituent (IC_50_=0.14 ± 0.05 µM). However, it is important to note that complex **1f** cannot fully inhibit the enzyme. Complex **1c** inhibits PL^Pro^ in a similar manner as complex **1a** with IC_50_ value of 0.54 ± 0.25 µM and complex **1g** is a slightly weaker inhibitor (IC_50_=1.66 ± 0.31 µM). In addition, it is important to note that the ligands themselves are again much weaker PL^Pro^ inhibitors compared to their zinc complexes (Supplementary Table 3). Our study has shown that substitution of parent pyrithione compound with selected groups has some influence on the biological response (enzyme inhibition) but none of the substituted compounds tested was much more effective. However, we believe that introduction of other groups might bring greater changes and should be tested in the future.

### *Ex vivo* SARS-CoV-2 entry and replication-competent assays

**Figure 3.**
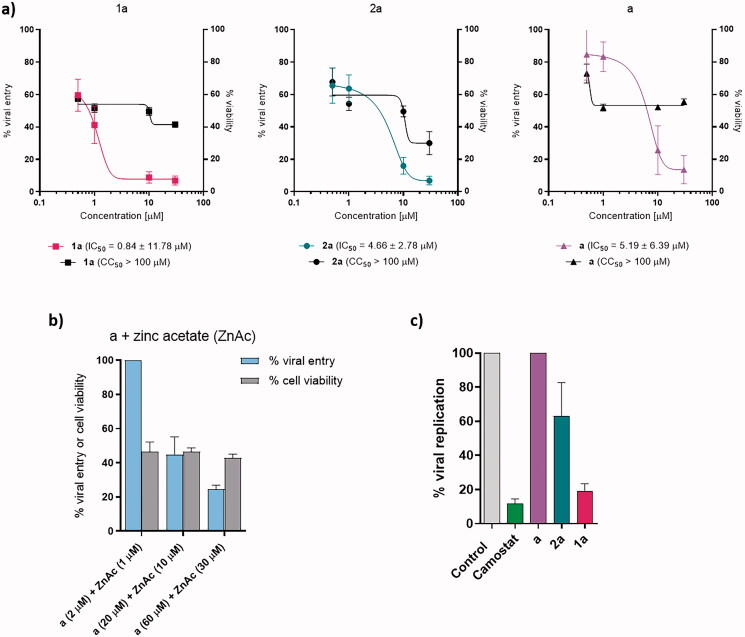
Viral entry, viral replication, and cell survival assays. (a) Viral entry assay of **a**, **1a**, and **2a** together with cell viability. Data are presented as mean ± S.E.M. (*n* = 3 independent experiments with technical triplicates for viral entry and duplicates for cell viability). (b) Viral entry assay after the application of pyrithione **a** and zinc acetate together with cell viability. Data are presented as mean ± S.E.M. (*n* = 1 with technical triplicates for viral entry and duplicates for cell viability). (c) Viral replication-competent assay for **a**, **1a**, and **2a**. Data are presented as mean ± S.E.M. (*n* = 1 with technical triplicates). Camostat was used as a positive control of viral inhibition.

Following the promising results of the enzymatic assays, we also wanted to test the efficacy of the selected compounds in an *ex vivo* physiological system of SARS-CoV-2 entry and infection, recently developed and derived from primary HLT^44^. Although some differences in the inhibition of cathepsin L and PL^Pro^ were observed for zinc complexes **1b**–**h** with various pyrithione derivatives, the differences were not significant compared to **1a**. Since the antimicrobial properties of zinc pyrithione **1a** have been well studied and this compound is also present in commercial shampoos, we decided to test complex **1a** in an *ex vivo* system for potential antiviral properties. In addition, ligand **a** was tested as a control and ruthenium complex **2a** was tested to investigate the importance of the metal core of the complex in inhibiting viral entry and replication (Figure 3).

The viral entry assay was used to determine how effective the compounds tested (**a**, **1a**, **2a**) were in preventing the entry of pseudoviral particles carrying the SARS-CoV-2 S protein into HLT cells at concentrations of 30 µM, 10 µM, 1 µM, and 0.5 µM (Figure 3(a)). Viral entry was inhibited in a concentration-dependent manner. At 30 µM, zinc and ruthenium complexes, **1a** and **2a**, respectively, had comparable effects with an inhibition of 93.12% and 93.16%, respectively, while ligand **a** showed an inhibition of 86.41%. The observed difference in inhibition between the complexes is more pronounced at 10 µM, where zinc complex **1a** inhibited viral penetration by 91.18%, ruthenium complex **2a** by 83.9% and ligand **a** by 74.34%. When ligand **a** was tested at 1 µM and 0.5 µM, it inhibited viral entry by 16.67% and 15.33%, respectively, while ruthenium complex **2a** showed an inhibition of 36.7% and 34.54%, respectively, and zinc complex **1a** showed 51.80% and 40.44% inhibition at the same concentrations. Thus, calculated IC_50_ values were 0.84 µM for **1a**, 4.66 µM for **2a**, and 5.19 µM for **a**. Similar to enzyme inhibition assays, ligand **a** was therefore found to be the worst inhibitor of viral entry, followed by ruthenium complex **2a** and zinc complex **1a**. Moreover, zinc also contributes to better inhibition of viral entry than ruthenium.

Besides, viral entry inhibition potential of the mixture of zinc acetate and pyrithione **a** was tested (molar ratio 1:2, c(zinc acetate)=1 µM, 10 µM, and 30 µM; Figure 3(b)). In all cases, zinc pyrithione **1a** was found to inhibit SARS-CoV-2 entry better than the above combination at all concentrations tested.

In addition to viral entry inhibition, the viability of HLT must also be taken into consideration. As expected, highest cell survival was observed at lowest tested concentration (0.5 µM) where cell survival reached 72.91%, 67.70%, and 57.39% for **a**, **2a**, and **1a**, respectively. Ligand pyrithione **a** at concentration 1–30 µM showed comparable cell viability of 51.91–55.51%. In the case of ruthenium complex **2a** at 30, 10, and 1 µM, cell viability was concentration-dependent, resulting in 30.02–54.26% of viable cells, while for zinc complex **1a** cell viability was 41.45–51.64%. Interestingly, cell viability remained constant (42.77–46.53%) when a combination of zinc acetate and pyrithione was tested at various concentrations.

Results for viral replication-competent assay are shown in Figure 3(c). Zinc complex **1a** inhibited 81.03% of SARS-CoV-2 replication at 30 µM and ruthenium complex **2a** prevented only 36.96% of viral replication. In addition, ligand **a** showed no effect on viral replication.

## Discussion

Metallodrugs hold a great potential as antiviral therapeutics^51^. During the pandemic, much attention was paid to zinc because of its antiviral properties. Zinc improves mucociliary clearance (removal of viral particles from the lungs) and strengthens the integrity of the respiratory epithelium. Importantly, increased Zn^2+^ concentrations may also improve antiviral immunity by upregulating IFNα and prevent inflammation by inhibiting NF-κB signalling. In addition, hypozincemia is associated with loss of smell and taste^11^^,^^16^^,^^17^^,^^19^ and is reported to lead to higher severity and mortality of COVID-19 patients^52^.

As mentioned in section “Introduction”, Zn^2+^ can indirectly inhibit SARS-CoV-2 targets such as ACE2 receptors, furin, RdRp, and M^Pro^. However, it is important to address the issue of zinc bioavailability. A randomised clinical trial suggests that oral treatment with zinc gluconate alone or in combination with ascorbic acid does not significantly shorten the duration of the COVID-19 symptoms^53^, whereas zinc sulphate tablets may improve survival in COVID-19 patients. However, the results are not clinically applicable due to various limitations of the study^54^. On the other hand, an ongoing clinical trial of intravenous administration of ZnCl_2_ has so far confirmed its safe use and shown an increase in serum zinc levels^55^^,^^56^. However, oral administration of the drug is much more patient-friendly and is preferred over intravenous use. Ionophores, molecules that enable the transport of metal ions across a lipid cell membrane, increase zinc uptake and facilitate zinc absorption, thus combating COVID-19^12^^,^^57–59^. In a multi-centre study testing zinc sulphate and hydroxychloroquine as potential treatments, increased hospital discharge and decreased in-hospital mortality were observed^60^. However, hydroxychloroquine as a therapeutic against COVID-19 should be considered with great caution as it may significantly increase the risk of cardiac arrhythmias. Similarly, a high risk of cardiac dysfunction has been reported for chloroquine^61–63^. Therefore, the choice of an appropriate chelating agent for zinc is of utmost importance.

Pyrithione **a** is a known ionophore with the ability to bind zinc ions, increase their intracellular concentration^64^ and as such has recognised antiviral activity against coronavirus (SARS-CoV), arterivirus, coxsackievirus, mengovirus, picornavirus, and rhinovirus^65^. A decade ago, te Velthuis et al. demonstrated that the combination of Zn^2+^ ions and pyrithione **a** effectively inhibited the replication of SARS-CoV via Zn^2+^ suppression of RdRp^25^. In addition, zinc pyrithione **1a** is an established antimicrobial agent used in commercial shampoos for dandruff^33^ treatment and exerts antifungal activity by damaging iron-sulphur clusters^32–34^. Recently, Maio et al. have suggested that Fe–S clusters act as cofactors of the SARS-CoV-2 RdRp enzyme and therefore represent another potential target to combat COVID-19^35^. Therefore, pyrithione **a** and its zinc complex **1a** have attracted much attention to combat SARS-CoV-2 during the COVID-19 pandemic because of the aforementioned antimicrobial effects. However, no systematic study on zinc-pyrithione complexes was reported so far.

In the present study, we focussed on two previously less studied but still very important targets of SARS-CoV-2, namely cathepsin L, which is involved in viral entry, and the protease PL^Pro^, which is involved in viral replication. Inhibition of PL^Pro^ is often unjustly overlooked in the development of potential multimodal SARS-CoV-2 therapeutics, and thus this target should also be considered in the development of SARS-CoV-2 antiviral agents^66^. Cathepsin L is a cysteine protease that cleaves the spike protein once SARS-CoV-2 is in the endolysosome within the host cell, and as such allows internalisation of the virus in a manner other than TMPRSS2 and furin. Cathepsin L has been reported to be overexpressed after infection with SARS-CoV-2, allowing further progression of infection and leading to a vicious cycle: the more cathepsin L is expressed, the more severe the progression of COVID-19. Therefore, cathepsin L is a promising target to prevent SARS-CoV-2 internalisation and further replication^67^^,^^68^. In this study, we demonstrated that zinc pyrithione **1a** is a promising cathepsin L inhibitor with an IC_50_ value in the micromolar range (1.88 ± 0.49 µM). Although some differences in cathepsin L inhibitory activity were observed between the tested analogous zinc complexes **1b**–**h**, the methyl/methoxy substituents on pyrithione and its scaffold extension did not have a major influence. On the other hand, the binding of the pyrithione ligands **a**–**h** to the zinc core contributes significantly to the inhibition of cathepsin L and PL^Pro^ since the ligands themselves are only weak inhibitors (Supplementary Table 3). Remarkably, ruthenium pyrithione complex **2a** was also a less potent cathepsin L inhibitor (IC_50_=124 ± 23 µM), indicating the importance of metal core selection. Similar behaviour of the tested compounds was observed for the cysteine protease PL^Pro^, with zinc complexes being the most efficient PL^Pro^ inhibitors, and all exhibiting IC_50_ values in the submicromolar range (e.g. IC_50_(**1a**)=0.50 ± 0.07 µM), followed by ruthenium complex **2a** (IC_50_=14.52 ± 2.49 µM) and ligands (IC_50_ not determined). Similar to our results of PL^Pro^ inhibition, zinc pyrithione **1a** also effectively inhibited both proteases from SARS-CoV, i.e. PL^Pro^ with an IC_50_ value of 3.7 µM^28^ and M^Pro^ with a *K*_i_ value of 0.17 µM^26^^,^^27^. In addition, zinc pyrithione **1a** also inhibited SARS-CoV-2 M^Pro^ with IC_50_ values in the submicromolar range (IC_50_=0.9687 µM^29^ and 0.1 µM^30^). The promising inhibition of cathepsin L and PL^Pro^ ^69^ by zinc complexes, but not by ligands alone, could be attributed to the thiophilic character of zinc^59^ and its binding to cysteine residues in the active sites of the aforementioned proteases. An interesting study discussing the influence of zinc coordination sphere on the reactivity of the metal centre showed, that certain type of a ligand affects Lewis acidity as well as hard/soft character of the zinc. In particular, thiolate ligands soften the zinc core and enable more interactions with functional groups such as amines or amides^70^, which are present also in the catalytic triade of the cathepsin L (Cys138–His276–Asn300)^71^ and PL^Pro^ (Cys111, His272, and Asp286)^72^. It is worth noting that Wang et al. recently reported that a mixture containing colloidal bismuth subcitrate can significantly inactivate viral cysteine proteases by binding the key cysteine residue in the active sites of PL^Pro^ and M^Pro^, thus providing a potential treatment to combat SARS-CoV-2^73^. On the other hand, the thio group of the pyrithione ligand could also interact with Cys residues. Indeed, it has been suggested that thiocarbonyl-containing compounds could be effective PL^Pro^ (SARS-CoV) inhibitors due to covalent bond between the thio moieties of the inhibitor and the Cys residue in the active site of the enzyme^74^. All encouraging data from the literature as well as our new promising findings indicate that zinc pyrithione complexes are potential zinc-containing drugs for COVID-19 oral treatment. In the past, the approach of one drug having effect on one target of one disease was the main strategy in drug development. In recent years, however, the polypharmacologic approach, in which a drug acts on multiple targets involved in disease pathways, has gained acceptance. Such a strategy would provide more effective therapeutics to patients^75^. Zinc pyrithione, with its proven inhibition of RdRp, M^Pro^, PL^Pro^, and cathepsin L, meets the criteria for polypharmacology. However, it is also important that the potential drug candidate does not interfere with off-targets, which would lead to adverse effects. Considering that zinc pyrithione **1a** is already approved for clinical use and its biological effects have been extensively studied, complex **1a** was selected as a promising candidate for further investigation of its anti-SARS-CoV-2 entry and replication properties in a highly physiological *ex vivo* system^44^.

The *ex vivo* system is composed of primary HLT and offers many advantages over immortalised cells derived, for instance, from kidney or colon cell lines. The HLT system provides type II alveolar cells, along with other heterogeneous cell components from lung tissue that are not subjected to any differentiation processes. Importantly, the HLT system also expresses factors that are required for SARS-CoV-2 entry, such as ACE2, CD147, TMPRSS2, and AXL. Indeed, zinc pyrithione **1a** inhibited viral entry at all concentrations tested, i.e. 0.5 µM, 1 µM, 10 µM, and 30 µM, with an IC_50_ of 0.84 µM. On the other hand, pyrithione **a** and ruthenium complex **2a** showed promising inhibition only at higher concentrations tested (10 and 30 µM), whereas at 0.5–1 µM only 36.4% and 34.54% of viral entry was prevented by ruthenium complex **2a** and 16.67% and 15.33% by pyrithione **a**. Since pyrithione **a** is known to be a zinc ionophore, the combination of zinc acetate and pyrithione **a** in molar ratio 1:2 was also tested. It was found that the combination of zinc and pyrithione produced weaker inhibition than the zinc pyrithione complexes at comparable concentrations tested. Moreover, when 1 µM zinc acetate and 2 µM pyrithione were applied to the cells, viral entry was not inhibited at all.

In addition, the *ex vivo* system used also supports the investigation of SARS-CoV-2 replication inhibition. Pyrithione **a** itself did not inhibit the replication of SARS-CoV-2. Again, zinc pyrithione **1a** was found to be the most active among the compounds tested, providing 81.03% inhibition of SARS-CoV-2 replication at 30 µM, followed by ruthenium complex **2a**, which was only able to suppress replication by 36.96%.

The biological activity of the compound is, of course, a prerequisite for its possible use as a therapeutic agent. However, the efficacy of the drug in the body depends on many parameters, including the physicochemical and ADME (absorption, distribution, metabolism, and excretion) properties of the drug candidate, which influence the pharmacokinetic and pharmacodynamic properties of the potential drug. The descriptors incorporated into the SwisADME tool showed that zinc pyrithione **1a** has promising properties that are favourable for drug design (Supplementary Figure 23)^76^. Generally, the compounds with log*P* values in the range of 1–4 are considered to be suitable for use as an oral drug. As such predicted logPo/w value of 1.65 for complex **1a** meets this requirement. Based on the calculations, complex **1a** is expected to have high gastrointestinal absorption without permeation through the blood–brain barrier. Zinc pyrithione **1a** also meets the standards of Lipinski's rule of five and thus represents a “drug-like” compound with favourable bioavailability. One parameter that should be considered with a little more caution is the solubility of complex **1a**, which should be taken into account when preparing a potential final peroral formulation^76^^,^^77^. Nowadays, however, advances in technological modifications allow many innovative delivery systems to improve oral drug bioavailability, e.g. nanoformulations, liposomes, co-crystallisation, cyclodextrins, polymers, etc.^78^^,^^79^.

In summary, zinc pyrithione **1a** shows a potential to combat SARS-CoV-2. The results obtained point that coordination of two pyrithione (**a**) ligands specifically to the zinc core is essential for the desired antiviral activity. Although some modifications should be considered to achieve better cell viability, overall complex **1a** holds great potential in the fight against SARS-CoV-2 because of its demonstrated multistep antiviral inhibitory properties. The multi-target approach to drug design has become a common strategy to avoid the possible development of inefficient drugs against SARS-CoV-2 infections as well. No therapeutic is universally effective, so broadening the spectrum of options in the fight against COVID-19 is of paramount importance, especially due to rapidly emerging mutations of the SARS-CoV-2 virus. To date, zinc pyrithione **1a** has shown excellent inhibition of the enzyme cathepsin L, which is involved in SARS-CoV-2 entry, as well as inhibition of M^Pro^ and PL^Pro^, enzymes involved in SARS-CoV-2 replication. Moreover, *ex vivo* studies have confirmed the potential of zinc pyrithione **1a** against SARS-CoV-2 entry and replication, expanding existing knowledge of its antiviral potential. Since zinc pyrithione **1a** is already an established antimicrobial agent commercially available, further research activities for possible repurposing of this zinc-containing agent for oral at-home SARS-CoV-2 patient treatment might be accelerated in the direction for potential clinical use.^80^

## Supplementary Material

Supplemental Material

## Data Availability

The additional data that support the findings of this study are available from the corresponding author upon reasonable request. The manuscript together with SI has been uploaded to bioRxiv preprint server with https://doi.org/10.1101/2022.03.03.482819.^80^
