# Correction

**DOI:** 10.1080/14756366.2022.2126157

**Published:** 2022-09-20

**Article title:** Zinc Pyrithione is a Potent Inhibitor of PLPro and Cathepsin L Enzymes with Ex Vivo Inhibition of SARS-CoV-2 Entry and Replication

**Authors:** Jerneja Kladnik, Ana Dolinar, Jakob Kljun, David Perea, Judith Grau-Expósito, Meritxell Genescà, Marko Novinec, Maria J. Buzon, Iztok Turel

**Journal:** *Journal of Enzyme Inhibition and Medicinal Chemistry*

**Bibliometrics:** Volume 37, Number 1, pages 2158–2168

**DOI:** 10.1080/14756366.2022.2108417

1) When this paper has been published online, the Graphical Abstract is missing. Below is the Graphical Abstract of the manuscript.


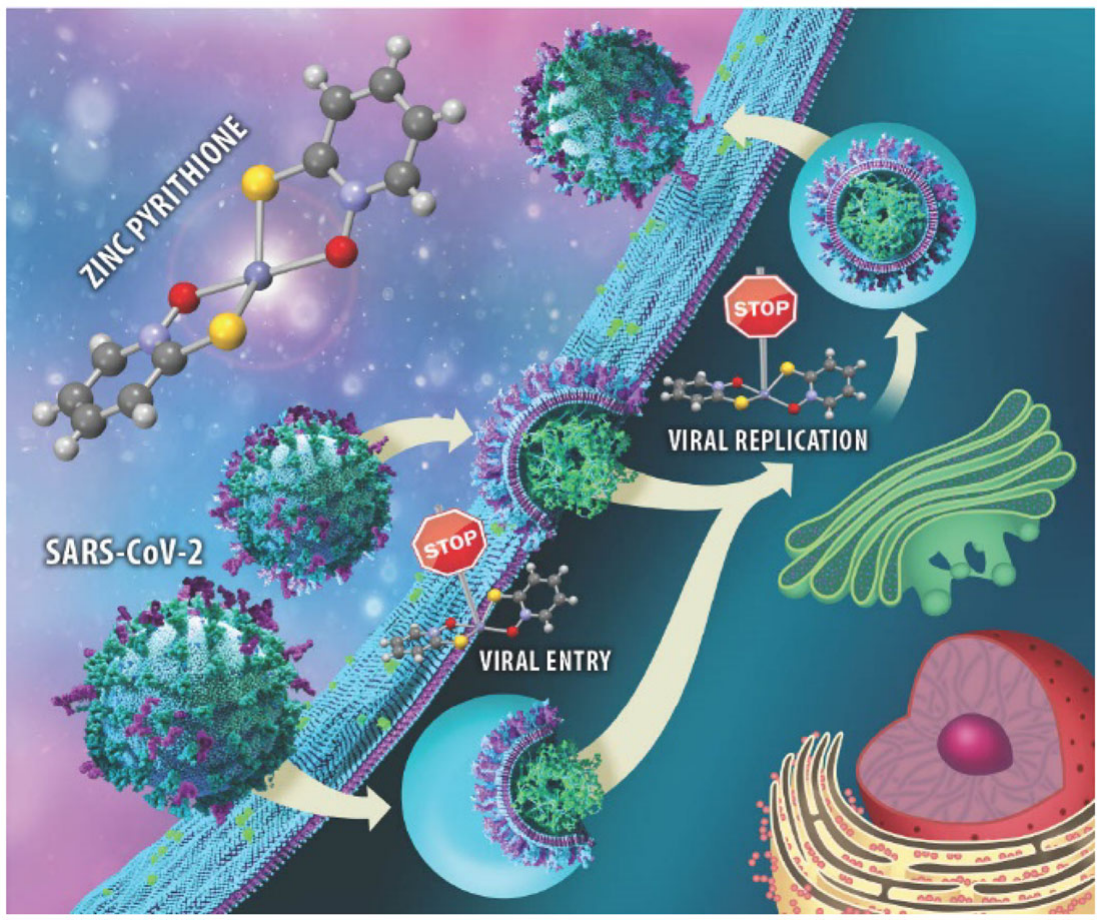


**2) The correct and updated version of the Acknowledgement is as follows**

The authors acknowledge EATRIS Academic Collaboration and their COVID-19 Fast Response Service for the coordination of new collaboration establishment between research group of Prof. Dr. I.Turel and Dr. Marıa J. Buzon. We would also like to thank Masa Masic for the help with the synthesis and Tja_sa Rijavec for the crystallization of complex **1d.** Schematic representation of the zinc pyrithione antiviral activity (Table of Content Graphic) was created by the artist Simon Kajtna.

