# Supplementary material for: Zinc pyrithione is a potent inhibitor of PLPro and cathepsin L enzymes with ex vivo inhibition of SARS-CoV-2 entry and replication
Source: J Enzyme Inhib Med Chem. 2022 Aug 9;37(1):2158–68. doi: 10.1080/14756366.2022.2108417 (PMC9367663; doi:10.1080/14756366.2022.2108417)
Supplement: Supplemental Material [file IENZ_A_2108417_SM6567.pdf]

Supplementary Information

Zinc Pyrithione is a Potent Inhibitor of PL^{Pro} and Cathepsin L Enzymes with *Ex Vivo* Inhibition of SARS-CoV-2 Entry and Replication

Jerneja Kladnik[a], Ana Dolinar[a], Jakob Kljun[a], David Perea[b], Judith Grau-Expósito[b], Meritxell Genescà[b], Marko Novinec[a], Maria J. Buzon[b*], Iztok Turel[a*]

[a] *Faculty of Chemistry and Chemical Technology, University of Ljubljana, Večna pot 113, SI-1000 Ljubljana, Slovenia*

[b] *Infectious Diseases Department, Vall d'Hebron Research Institute (VHIR), Hospital Universitari Vall d'Hebron, Universitat Autònoma de Barcelona, VHIR Task Force COVID-19, Barcelona, Spain*

* Corresponding authors: mariajose.buzon@vhir.org, iztok.turel@fkkt.uni-lj.si

Table of Contents

1. General information 2
2. Synthesis and Characterization 3
3. Crystal structures 6
4. NMR spectra 10
5. UV-vis and NMR stability 18
6. Enzyme assays 23
7. Prediction data for the key parameters in drug design 26
8. References 27

1. General information

Ligand pyrithione **a**, starting materials and other reagents as well as solvents for the synthesis of ligands **b**–**h**, complexes **1a**–**h** and **2a**, were purchased from commercial suppliers (Fluorochem, Alfa Aesar, Riedel-de-Haën, Merck) and used as received without further purification.

The progress of the reaction was monitored by thin layer chromatography using pre-coated TLC sheets ALUGRAM® SIL G/UV254 (Macherey–Nagel) visualised with UV lamp (254 nm). The column chromatography of the ligands was carried out on Merck silica gel 60 (35–70 mm). ^{1}H NMR spectra for compound characterization were acquired on Bruker Avance III 500 spectrometer at room temperature at 500 MHz. ^{1}H NMR stability spectra were obtained on NMR Bruker AvanceNeo 600 MHz spectrometer at room temperature at 600 MHz. All chemical shifts (δ) in the spectra of the complexes are referenced to the residual peaks of deuterated solvent $(CD_3)_2SO$, $CDCl_3$ and D_2O at 2.50 (referenced to the central line of a quintet), 7.26 and 4.79 ppm, respectively and are reported in ppm. Coupling constants (*J*) are reported in Hz. Spectra were processed in MestReNova 11.0.4. The multiplicity of the signals is abbreviated as s – singlet, d – doublet, t – triplet and m – multiplet. IR spectra were obtained on a Bruker FTIR Alpha Platinum ATR spectrometer. High resolution mass spectrometry was performed on an Agilent 6224 Accurate Mass TOF LC/MS system. Elemental analysis (C, H, N) was carried out on a PerkinElmer 2400 II instrument. UV-vis spectroscopy was performed on a PerkinElmer LAMBDA 750 UV/Vis/near-IR spectrophotometer. For X-ray structural analysis, single crystals were surrounded with silicon grease, mounted onto the tip of glass fibres and transferred to the goniometer head in the liquid nitrogen cryostream (150(2) K). Data were collected on a SuperNova diffractometer equipped with Atlas detector using CrysAlis software with monochromated Mo Kα (0.71073 Å).[1] The initial structural models were obtained via direct methods using the Olex2 graphical user interface[2] implemented in SHELXT. A full-matrix least-squares refinement on F^2 magnitudes with anisotropic displacement parameters for all non-hydrogen atoms using Olex2 or SHELXL-2018/3 was employed.[2,3] All non-hydrogen atoms were refined anisotropically, while the hydrogen atoms were placed at calculated positions and further treated as riding on their parent atoms. Figures depicting the structures were prepared with Mercury.[4] The crystal structures have been submitted to the CCDC and have been allocated the deposition numbers 2143703-2143706. These data are provided free of charge by The Cambridge Crystallographic Data Centre.[5]

Synthetic fluorogenic substrates benzyloxycarbonyl-Leu-Arg-7-amino-4-methylcoumarin (Z-LR-AMC; Cat. No. 4034611) and benzyloxycarbonyl-Arg-Leu-Arg-Gly-Gly-7-amino-4-methylcoumarin (Z-RLRGG-AMC; Cat. No. 4027158) were purchased from Bachem. Ni-affinity column HisTrap was purchased from GE Healthcare.

OD_{600} measurements were obtained with Varian Cary 50 Bio spectrophotometer (Agilent). Protein elution was performed on ÄKTA FPLC system (GE Healthcare). Enzymatic kinetic measurements were performed on a PerkinElmer LS55 fluorescence spectrometer with PTP-1 Fluorescence Peltier System and Peltier Controlled Fluid Circulator (PCB1500).

2. Synthesis and Characterization

Ligands **b**–**g** were synthesized as previously reported.[6–8]

Newly prepared ligand **h** was synthesized according to the same procedure as ligands **b**–**e**.

*2-Bromo-3-methoxypiridine N-oxide.*The starting material 2-bromo-3-methoxypyridine (2.659 mmol, 1 mol. equiv.) was dissolved in dichloromethane (30 mL) and *m*-chloroperoxybenzoic acid (2 mol. equiv., 70% purity) was added to the solution and stirred over the night at room temperature. Next day, reaction mixture was first washed with $Na_2S_2O_3$ aqueous solution (0.5 M, 1 x 50 mL; water phase WP1) and then with saturated $NaHCO_3$ aqueous solution (1 x 25 mL; WP2), obtaining organic phase OP1. WP1 was further extracted with dichloromethane (1 x 50 mL), which was consequently washed with saturated $NaHCO_3$ aqueous solution (1 x 50 mL). The latter water phase was then extracted with dichloromethane (2 x 50 mL), obtaining OP2. WP2 was extracted with dichloromethane (2 x 25 mL), obtaining organic phase OP3. OP1–3 were joined together, dried over Na_2SO_4, filtered, dichloromethane was evaporated and the oily residue was purified by column chromatography (stationary phase: silica gel, mobile phase: 2% MeOH/DCOM). After the evaporation of the selected fractions white solid was obtained.

Yield: 65%. ^{1}H NMR (500 MHz, $CDCl_3$): δ = 8.09 (dd, 1H, J = 6.6, 1.2 Hz, Ar–*H*), 7.15 (dd, 1H, J = 8.6, 6.6 Hz, Ar–*H*), 6.79 (dd, 1H, J = 8.6, 1.1 Hz, Ar–*H*), 3.96 (s, 3H, Ar–OCH_3) ppm. ESI-HRMS (CH_3CN): *m/z* calcd for $[C_6H_7BrNO_2]^+$: 203.9655; found: 203.9656. Elemental analysis calcd (%) for $C_6H_6BrNO_2$: C, 35.32; H, 2.96; N, 6.87; found (%): C, 35.51; H, 2.92; N, 7.03.

*1-hydroxy-3-methoxypyridine-2(1H)-thion (**h**).* 2-Bromo-3-methoxypiridine *N*-oxide (100 mg) was dissolved in saturated $NaSH_{(aq)}$ solution (10 mL) together, followed by the addition of distilled water (10 mL). Orange solution was stirred overnight at the room temperature. Next day the solution was acidified with $HCl_{(aq)}$ (4 M) and extracted with $CHCl_3$ (2 x 30 mL). The combined organic layers were dried over Na_2SO_4 and filtered followed by solvent evaporation. Yellow solid was triturated with acetone (5 mL), by-product S_8 was filtered off and the mother liquor was evaporated. To ensure required purity of the ligand for the synthesis of the zinc complex, ligand **h** was purified by column chromatography (stationary phase: SiO_2; mobile phase: hexane/ethyl acetate=7/3). To the selected fractions mobile phase was evaporated and yellow solid was dried over night at 45 ºC.

Yield: 86%. ^{1}H NMR (500 MHz, $CDCl_3$): δ = 12.33 (bs, 1H, N–O*H*), 7.85 (dd, 1H, J = 6.6, 1.3 Hz, Ar–*H*), 6.80–6.71 (m, 2H, Ar–*H*), 3.96 (s, 3H, Ar–OCH_3) ppm. ESI-HRMS (CH_3CN): *m/z* calcd. for $[C_6H_8NO_2S]^+$: 158.0270; found: 158.0269. Elemental analysis calcd. (%) for $C_6H_7NO_2S$: C, 45.85; H, 4.49; N, 8.91; found (%): C, 45.78; H, 4.53; N, 8.95.

Zinc complexes **1a**–**h** have been prepared according to the following modified procedure.[9] Appropriate ligand **a**–**h** (0.786 mmol, 2 mol. equiv.) was dissolved in methanol (10 mL) to which 1M NaOH aqueous solution was added dropwise to obtain pH ~ 8. Then, aqueous solution (10 mL) of $Zn(CH_3COO)_2 \cdot 2H_2O$ (0.393 mmol, 1 mol. equiv.) was added to the solution of the ligand upon which white precipitate appeared immediately. Suspension was further stirred for 1 h at room temperature. White solid was filtered off under reduced pressure and first washed with methanol (10 mL) to eliminate a by-product CH_3COONa and additionally with diethyl ether (10 mL). Obtained zinc complexes **1a**–**h** were left to dry overnight at 45 ºC.

[Zn(II)(1-hydroxypyridine-2(1*H*)-thionato)$_2$] (**1a**).

Yield: 89%. ^{1}H NMR (500 MHz, $(CD_3)_2SO$)): δ = 8.43 (dd, 2H, *J* = 6.6, 1.2 Hz, Ar–*H* a), 7.61 (dd, 2H, *J* = 8.2, 1.8 Hz, Ar–*H* a), 7.28–7.22 (m, 2H, Ar–*H* a), 7.01 (td, 2H, *J* = 7.0, 1.8 Hz, Ar–*H* a) ppm. ESI-HRMS (CH_3CN): *m/z* calcd for $[C_{10}H_9N_2O_2S_2Zn]^+$: 316.9391; found: 316.9391. Elemental analysis calcd (%) for $C_{10}H_8N_2O_2S_2Zn$: C, 37.81; H, 2.54; N, 8.82; found: C, 37.59; H, 2.50; N, 8.59. IR selected bands (ATR): ṽ = 3102, 1457, 1199, 1147, 1087, 821, 762, 703, 582, 564 cm^{-1}.

[Zn(II)(1-hydroxy-3-methylpyridine-2(1*H*)-thionato)$_2$] (**1b**).

Yield: 87%. ^{1}H NMR (500 MHz, $CDCl_3$): *δ* = 8.26 (dd, 2H, *J* = 6.6, 0.6 Hz, Ar–*H* b), 7.24–7.20 (m, 2H, Ar–*H* b), 6.84 (t, 2H, *J* = 7.0 Hz, Ar–*H* b), 2.52 (s, 6H, Ar–C*H*$_3$ b) ppm. ESI-HRMS (CH_3CN): *m/z* calcd for $[C_{12}H_{13}N_2O_2S_2Zn]^+$: 344.9704; found: 344.9702. Elemental analysis calcd (%) for $C_{12}H_{12}N_2O_2S_2Zn$: C, 41.69; H, 3.50; N, 8.10; found: C, 41.34; H, 3.40; N, 8.00. IR selected bands (ATR): ṽ = 3073, 1403, 1206, 1146, 1134, 782, 702, 630, 565 cm^{-1}.

[Zn(II)(1-hydroxy-4-methylpyridine-2(1*H*)-thionato)$_2$] (**1c**).

Yield: 50%. ^{1}H NMR (500 MHz, $CDCl_3$): δ = 8.16 (d, 2H, *J* = 6.8 Hz, Ar–*H* c), 7.53 (dd, 2H, *J* = 1.6, 0.6 Hz, Ar–*H* c), 6.72 (dd, 2H, *J* = 6.8, 2.4 Hz, Ar–*H* c), 2.30 (s, 6H, Ar–C*H*$_3$ c) ppm. ESI-HRMS (CH_3CN): *m/z* calcd for $[C_{12}H_{13}N_2O_2S_2Zn]^+$: 344.9704; found: 344.9701. Elemental analysis calcd (%) for $C_{12}H_{12}N_2O_2S_2Zn$: C, 41.69; H, 3.50; N, 8.10; found: C, 41.53; H, 3.50; N, 7.97. IR selected bands (ATR): ṽ = 3072, 1614, 1469, 1197, 1138, 1085, 809, 780, 602, 488 cm^{-1}.

[Zn(II)(1-hydroxy-5-methylpyridine-2(1*H*)-thionato)$_2$] (**1d**).

Yield: 72%. ^{1}H NMR (500 MHz, $CDCl_3$): δ = 8.14 (s, 2H, Ar–*H* d), 7.61 (d, 2H, *J* = 8.5 Hz, Ar–*H* d), 7.07 (dd, 2H, *J* = 8.5, 1.4 Hz, Ar–*H* d), 2.26 (s, 6H, Ar–C*H*$_3$ d) ppm. ESI-HRMS (CH_3CN): *m/z* calcd for $[C_{12}H_{13}N_2O_2S_2Zn]^+$: 344.9704; found: 344.9705. Elemental analysis calcd (%) for $C_{12}H_{12}N_2O_2S_2Zn$: C, 41.69; H, 3.50; N, 8.10; found: C, 41.39; H, 3.38; N, 8.08. IR selected bands (ATR): ṽ = 3056, 1474, 1372, 1149, 1090, 810, 739, 664, 592, 548 cm^{-1}.

[Zn(II)(1-hydroxy-6-methylpyridine-2(1*H*)-thionato)$_2$] (**1e**).

Yield: 54%. ^{1}H NMR (500 MHz, $CDCl_3$): δ = 7.63 (dd, 2H, *J* = 8.3, 1.5 Hz, Ar–*H* e), 7.10 (t, 2H, *J* = 7.7 Hz, Ar–*H* e), 6.85 (dd, 2H, *J* = 7.7, 1.5 Hz, Ar–*H* e), 2.61 (s, 6H, Ar–C*H*$_3$ e) ppm. ESI-HRMS (CH_3CN): *m/z* calcd for $[C_{12}H_{13}N_2O_2S_2Zn]^+$: 344.9704; found 344.9702. Elemental analysis calcd (%) for $C_{12}H_{12}N_2O_2S_2Zn$: C, 41.69; H, 3.50; N, 8.10; found: C, 41.26; H, 3.39; N, 7.97. IR selected bands (ATR): ṽ = 3072, 1557, 1461, 1373, 1200, 1184, 889, 770, 646, 411 cm^{-1}.

[Zn(II)(2-hydroxyisoquinoline-1(2*H*)-thione)$_2$] (**1f**).

Yield: 93 %. ^{1}H NMR (500 MHz, $CDCl_3$): δ = 8.77–8.72 (m, 2H, Ar–*H* f), 8.22 (d, 2H, *J* = 7.2 Hz, Ar–*H* f), 7.78–7.73 (m, 2H, Ar–*H* f), 7.72–7.66 (m, 4H, Ar–*H* f), 7.30 (d, 2H, *J* = 7.2 Hz, Ar–*H* f) ppm. ESI-HRMS (CH_3CN): *m/z* calcd for $[C_{18}H_{13}N_2O_2S_2Zn]^+$: 416.9704; found: 416.9708. Elemental analysis calcd (%) for $C_{18}H_{12}N_2O_2S_2Zn$: C, 51.75; H, 2.90; N, 6.71; found: C, 51.48; H, 2.67; N, 6.60. IR selected bands (ATR): ṽ = 3088, 2970, 1547, 1334, 1306, 1199, 948, 789, 762, 746, 667 cm^{-1}.

[Zn(II)(1-hydroxyquinoline-2(1*H*)-thione)$_2$] (**1g**).

Yield: 70 %. ^{1}H NMR (500 MHz, $CDCl_3$): δ = 8.64 (d, 2H, *J* = 8.8 Hz, Ar–*H* g), 7.79–7.70 (m, 6H, Ar–*H* g), 7.66 (d, 2H, *J* = 8.8 Hz, Ar–*H* g), 7.53–7.48 (m, 2H, Ar–*H* g) ppm. ESI-HRMS (CH_3CN): *m/z* calcd for $[C_{18}H_{13}N_2O_2S_2Zn]^+$: 416.9704; found: 416.9704. Elemental analysis calcd (%) for

$C_{18}H_{12}N_2O_2S_2Zn$: C, 51.75; H, 2.90; N, 6.71; found: C, 51.87; H, 2.61; N, 6.72. IR selected bands (ATR): ṽ = 2970, 1738, 1600, 1561, 1306, 902, 808, 757, 654, 512 cm^{-1}.

[Zn(II)(1-hydroxy-3-methoxypyridine-2(1*H*)-thionato)$_2$] (**1h**).

Yield: 57%. ^{1}H NMR (500 MHz, $CDCl_3$): δ = 8.08 (dd, 2H, *J* = 6.5, 1.0 Hz, Ar–*H* h), 6.90–6.80 (m, 4H, Ar–*H* h), 3.98 (s, 6H, Ar–OCH_3 h) ppm. ESI-HRMS (CH_3CN): m/z calcd for $[C_{12}H_{13}N_2O_4S_2Zn]^+$: 376,9603; found: 376.9598. Elemental analysis calcd (%) for $C_{12}H_{12}N_2O_4S_2Zn$: C, 38.16; H, 3.20; N, 7.42; found: C, 37.97; H, 3.00; N, 7.47. IR selected bands (ATR): ṽ = 3108, 1553, 1441, 1424, 1292, 1222, 1067, 762, 696, 669 cm^{-1}.

Synthesis of ruthenium complex **2a** has been reported before by the Turel group.(7) Briefly, ruthenium precursor dichloro(*p*-cymene)ruthenium(II) dimer (0.0980 mmol, 1 mol equiv.), ligand **a** (2 mol. equiv.) and sodium methoxide (2 mol. equiv.) was dissolved in acetone (50 ml) in the round bottom flask and the reaction mixture was stirred at room temperature overnight. Next day, the solvent was evaporated, and the crude product was purified by column chromatography (stationary phase: SiO_2; mobile phase: 5% DCM/acetone). Mobile phase was evaporated to the selected fractions and the red soldi was obtained by precipitation using DCM/*n*-heptane solvent system.

3. Crystal structures

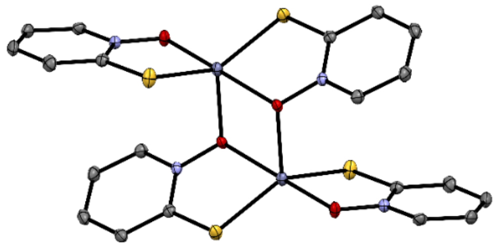

1a*

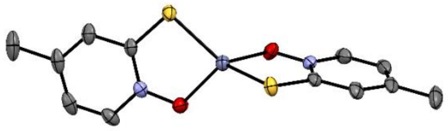

1c**

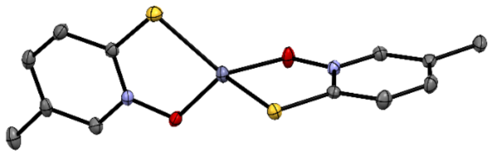

1d

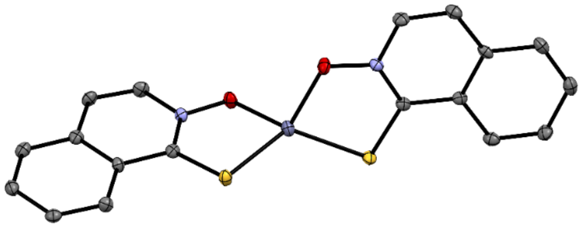

1f

Supplementary Figure 1: Crystal structure of complexes 1a, 1c, 1d and 1f. Ellipsoids are drawn at the 35% probability level.

*As the structure report for **1a** dates back to 1977,[10] we decided to re-measure data on a crystal sample of this compound to obtain high-quality data. **The reported structure is an ethanol solvate containing a heavily disordered solvent molecule. Crystals of **1c** were obtained by liquid diffusion of hexane into a chloroform solution. Single crystal X-ray diffraction shows the same structure even in the absence of ethanol. The coordinates of solvent molecules could not be reliably determined. Utilization of the solvent mask function in Olex2 (equivalent to Squeeze function from ShelX) shows the presence of a 41Å^3 void containing 8 e$^-$ corresponding most likely to approx. 1 water molecule per void.

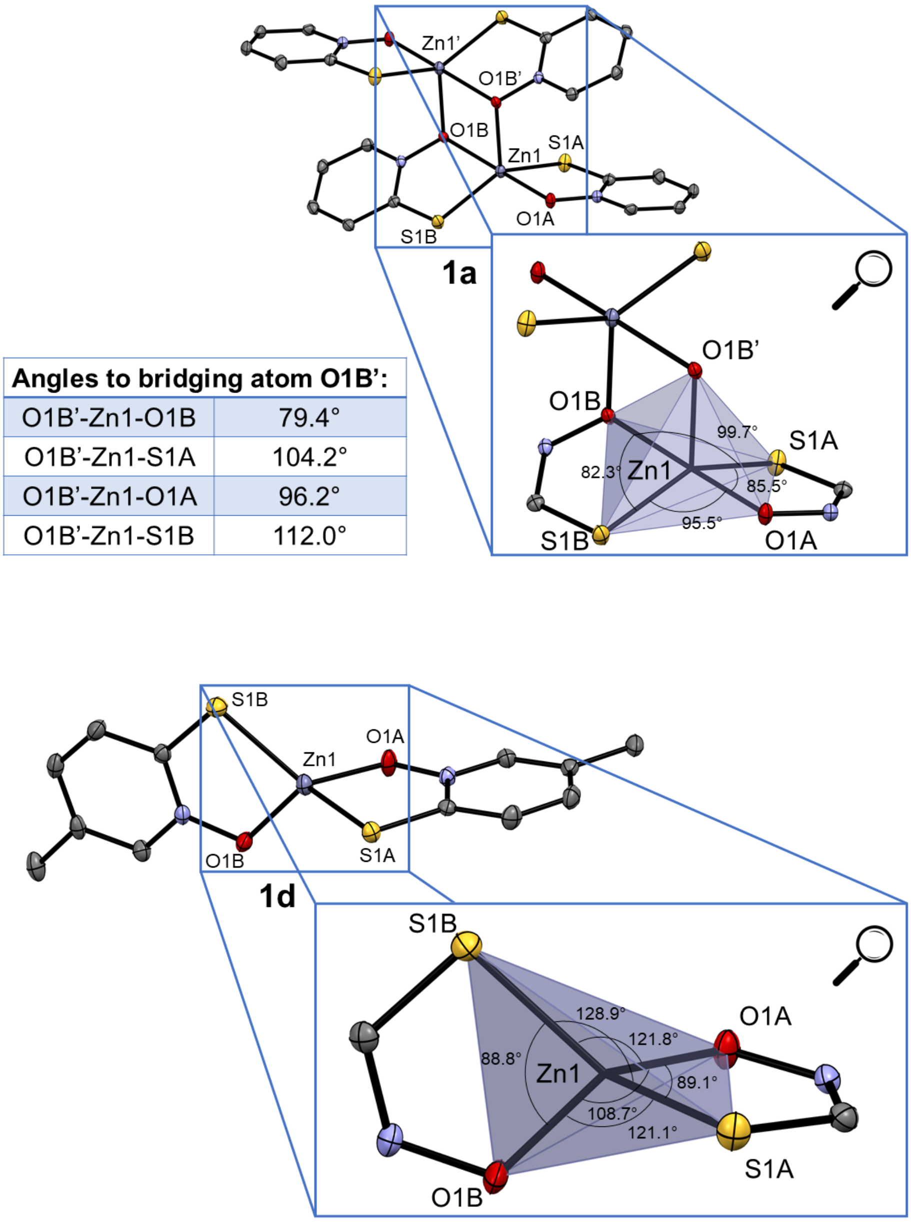

Angles to bridging atom O1B':	
O1B'-Zn1-O1B	79.4°
O1B'-Zn1-S1A	104.2°
O1B'-Zn1-O1A	96.2°
O1B'-Zn1-S1B	112.0°

Supplementary Figure 2: Crystal structure of complexes 1a and 1d (together with zoom of trigonal bipyramidal and tetrahedral coordination, respectively). Ellipsoids are drawn at the 35% probability level.

Supplementary Table 1: Crystallographic data for complexes 1a, 1c, 1d and 1d.

Compound	**1a**	**1c**	**1d**	**1f**
Dataset	moc470	mob156	mob128	moc457
CCDC dep. No.	2143706	2143704	2143703	2143705
Empirical formula	$C_{10}H_8N_2O_2S_2Zn$	$C_{12}H_{12}N_2O_2S_2Zn$	$C_{12}H_{12}N_2O_2S_2Zn$	$C_{18}H_{12}N_2O_2S_2Zn$
Formula weight	317.67	345.73	345.769	417.79
Temperature/K	150(2)	150	150	150
Crystal system	monoclinic	trigonal	monoclinic	triclinic
Space group	$P2_1/c$	R-3c	C2/c	P-1
a/Å	8.3347(4)	21.186(2)	27.7422(12)	7.0548(3)
b/Å	10.1317(4)	21.186(2)	7.2234(4)	7.0948(3)
c/Å	13.6447(6)	16.8962(9)	13.5512(6)	16.2539(9)
α/°	90	90	90	99.916(4)
β/°	96.748(4)	90	99.630(5)	95.707(4)
γ/°	90	120	90	98.714(4)
Volume/Å^3	1144.24(9)	6567.9(13)	2677.3(2)	785.53(7)
Z	4	18	8	2
ρ_{calc} [g/cm^3]	1.844	1.573	1.716	1.766
μ/mm^{-1}	2.499	1.966	2.144	1.844
F(000)	640.0	3168.0	1413.1	424.0
Crystal size/mm^3	0.25 × 0.25 × 0.25	0.7 × 0.1 × 0.1	1.0 × 0.9 × 0.8	0.1 × 0.05 × 0.03
Radiation	Mo Kα (λ = 0.71073)	Mo Kα (λ = 0.71073)	Mo Kα (λ = 0.71073)	Mo Kα (λ = 0.71073)
2Θ range for data collection/°	4.922 to 54.958	6.35 to 54.952	5.84 to 54.96	5.132 to 54.97
Index ranges	$-10 \le h \le 10$, $-13 \le k \le 13$, $-16 \le l \le 17$	$-18 \le h \le 22$, $-27 \le k \le 27$, $-21 \le l \le 19$	$-38 \le h \le 25$, $-9 \le k \le 10$, $-17 \le l \le 18$	$-8 \le h \le 9$, $-9 \le k \le 9$, $-21 \le l \le 21$
Reflections collected	11634	5691	8064	16062
Independent reflections	2609 [R_{int} = 0.0349, R_{sigma} = 0.0280]	1661 [R_{int} = 0.0245, R_{sigma} = 0.0207]	3075 [R_{int} = 0.0219, R_{sigma} = 0.0291]	3550 [R_{int} = 0.0513, R_{sigma} = 0.0428]
Data/restraints/parameters	2609/0/154	1661/0/88	3075/0/174	3550/0/226
Goodness-of-fit on F^2	1.055	1.251	1.050	1.137
Final R indexes [I>=2σ (I)]	R_1 = 0.0242, wR_2 = 0.0534	R_1 = 0.0446, wR_2 = 0.0923	R_1 = 0.0294, wR_2 = 0.0738	R_1 = 0.0695, wR_2 = 0.1942
Final R indexes [all data]	R_1 = 0.0302, wR_2 = 0.0567	R_1 = 0.0490, wR_2 = 0.0941	R_1 = 0.0350, wR_2 = 0.0775	R_1 = 0.0782, wR_2 = 0.1990
Largest diff. peak/hole / eÅ^{-3}	0.33/-0.37	0.43/-0.34	0.47/-0.34	2.58/-0.76*

*Comment of crystal structure of **1f:** The checkcif report contains B/C-type alerts due to residual peaks of electron density (Q1 2.58, Q2 2.56). These peaks cannot be refined by insertion of for example solvate water molecules. The absence of solvate water molecules is confirmed by CHN elemental analysis. The anomalies are attributed to lower quality of the single crystal. Despite that, all bond lengths and angles are within the value ranges typical for zinc complexes. At present this is the best data we were able to obtain.

Supplementary Table 2: Analysis of the available crystal structures of zinc-pyrithione complexes.

CCDC code / data set			**Zn1-O$_{terminal}$**	**Zn1-S**	**Zn1-O$_{bridge}$**	**O$_{bridge}$- Zn2**	**τ_5**
OXPTZN(10) (Zn-pth complex, chloroform solvate)	**1a**	dimer	2.0502	2.3106<br>2.315	2.1847	2.0925	0.59
OXPZND (Zn-pth complex)	**1a**	dimer	2.0494	2.3069<br>2.3075	2.1821	2.1215	0.52
MIKZEL	**1b**	dimer	2.049(3)	2.306(2)<br>2.295(2)	2.171(3)	2.127(4)	0.51
			**Zn-O**	**Zn-S**			**τ_4/τ_4'**
TETRAL[11] (Zn-(4Me-pth) complex, 1/6 ethanol solvate)	**1c**	monomer (symmetric)	1.968	2.275			0.78/0.74
Mob156 (Isostructural with TETRAL, EtOH atoms unassigned)	**1c**	monomer (symmetric)	1.973	2.270			0.78/0.74
Mob128 (Zn-(5Me-pth) complex)	**1d**	monomer	1.973(1)<br>1.968(1)	2.2859(7)<br>2.2729(8)			0.78/0.75
CAQCEC[12] (Zn-(6Me-pth) complex)	**1e**	monomer	1.983(2)<br>1.985(3)	2.2636(9)<br>2.2701(8)			0.76/0.70
Mob457 (Zn-(N-hydroxyisoquinoline-1-thione) complex)	**1f**	monomer	1.972(5)<br>1.965(4)	2.289(2)<br>2.286(1)			0.76/0.75

$\tau_5 = (\beta-\alpha)/(60°)$. For square-pyramidal geometry, the τ_5 value is 0, for ideal trigonal-bipyramidal geometry, the value of τ_5 is 1. $\tau_4 = [360°-(\alpha+\beta)]/(360°-2\cdot\theta)$; $\tau_4' = [(\beta-\alpha)/(360°-\theta)]+[(180°-\beta)/(360°-\theta)]$; α and β are the two greatest valence angles of coordination centre, θ is tetrahedral angle 109.471°. For ideal square-planar geometry τ_4 and τ_4' values are 0, for ideal tetrahedral geometry τ_4 and τ_4' values are 1.

See reference[13] for **τ_4**.
See reference[14] for **τ_4'**.
See reference[15] for **τ_5**.

4. NMR spectra

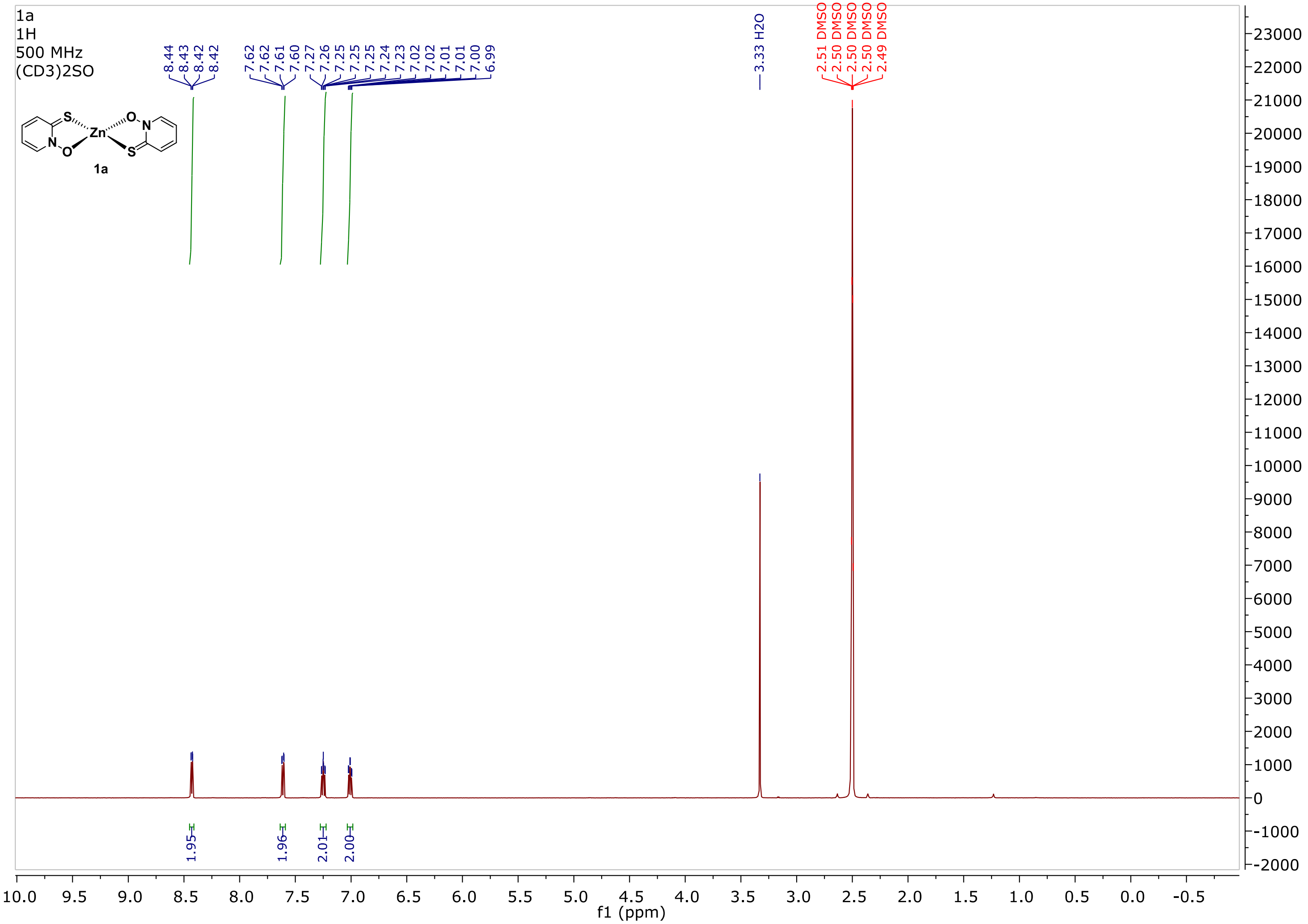

Supplementary Figure 3: ^{1}H NMR spectrum of **1a**.

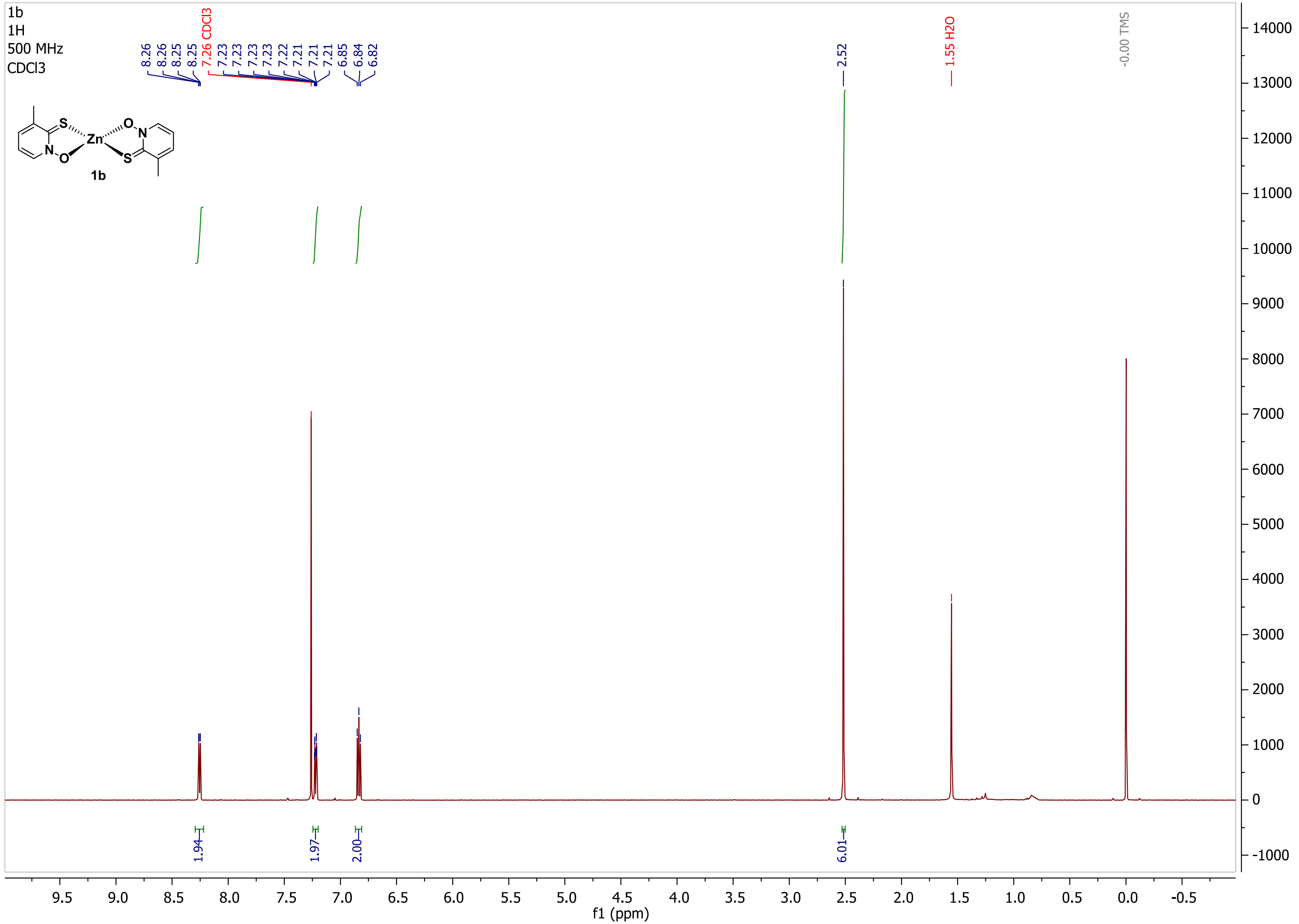

Supplementary Figure 4: ^{1}H NMR spectrum of **1b**.

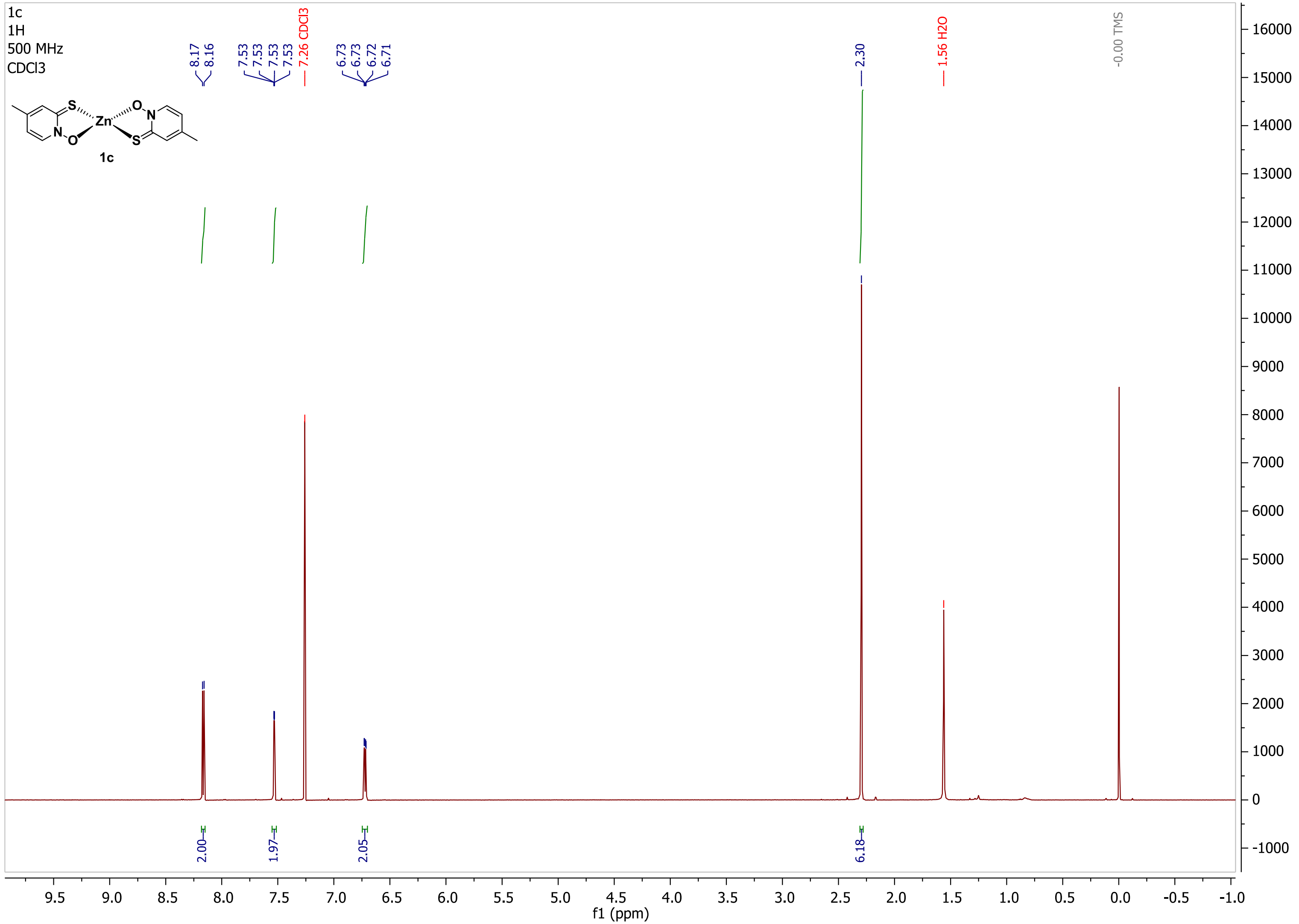

Supplementary Figure 5: ^{1}H NMR spectrum of **1c**.

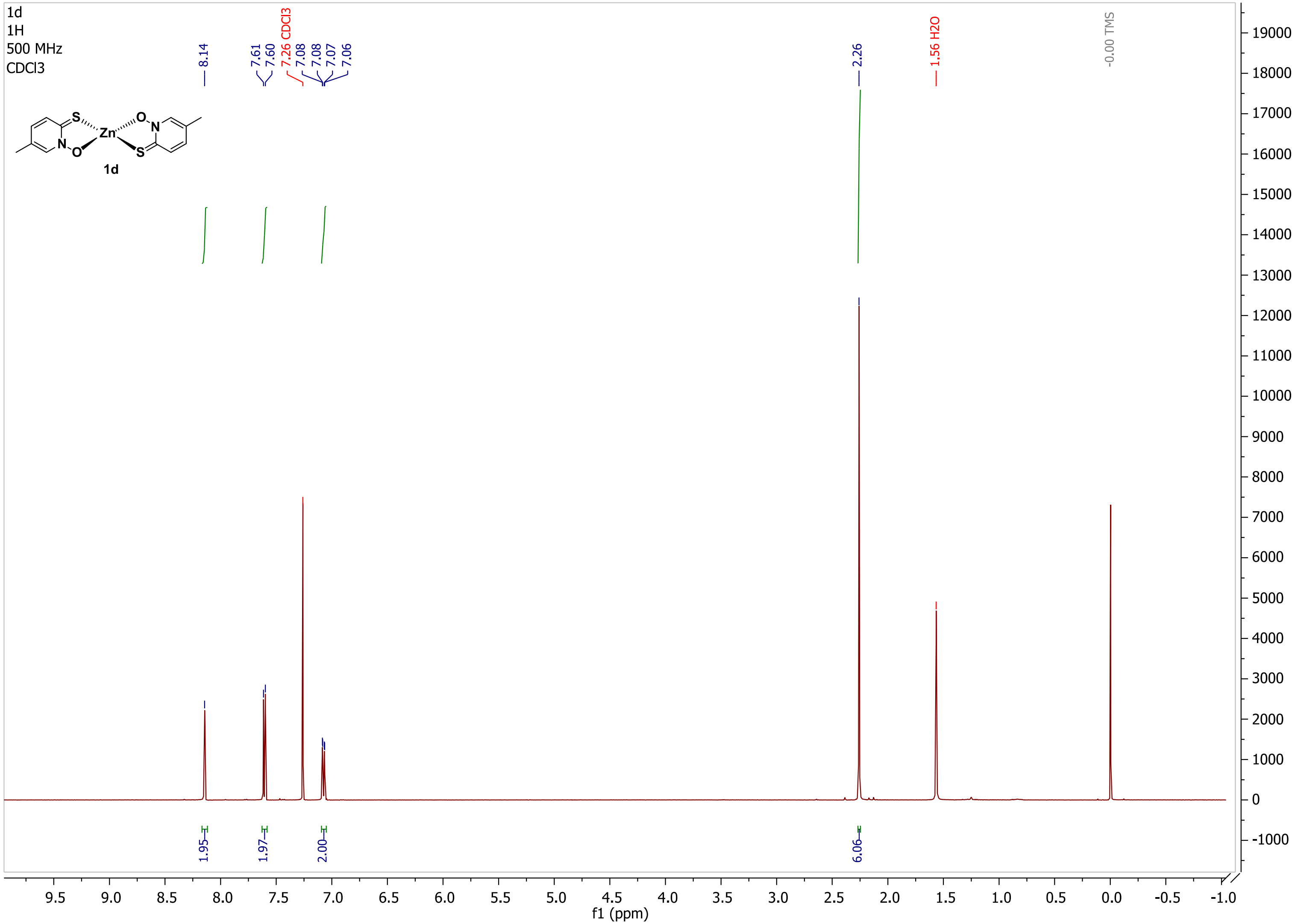

Supplementary Figure 6: ^{1}H NMR spectrum of **1d**.

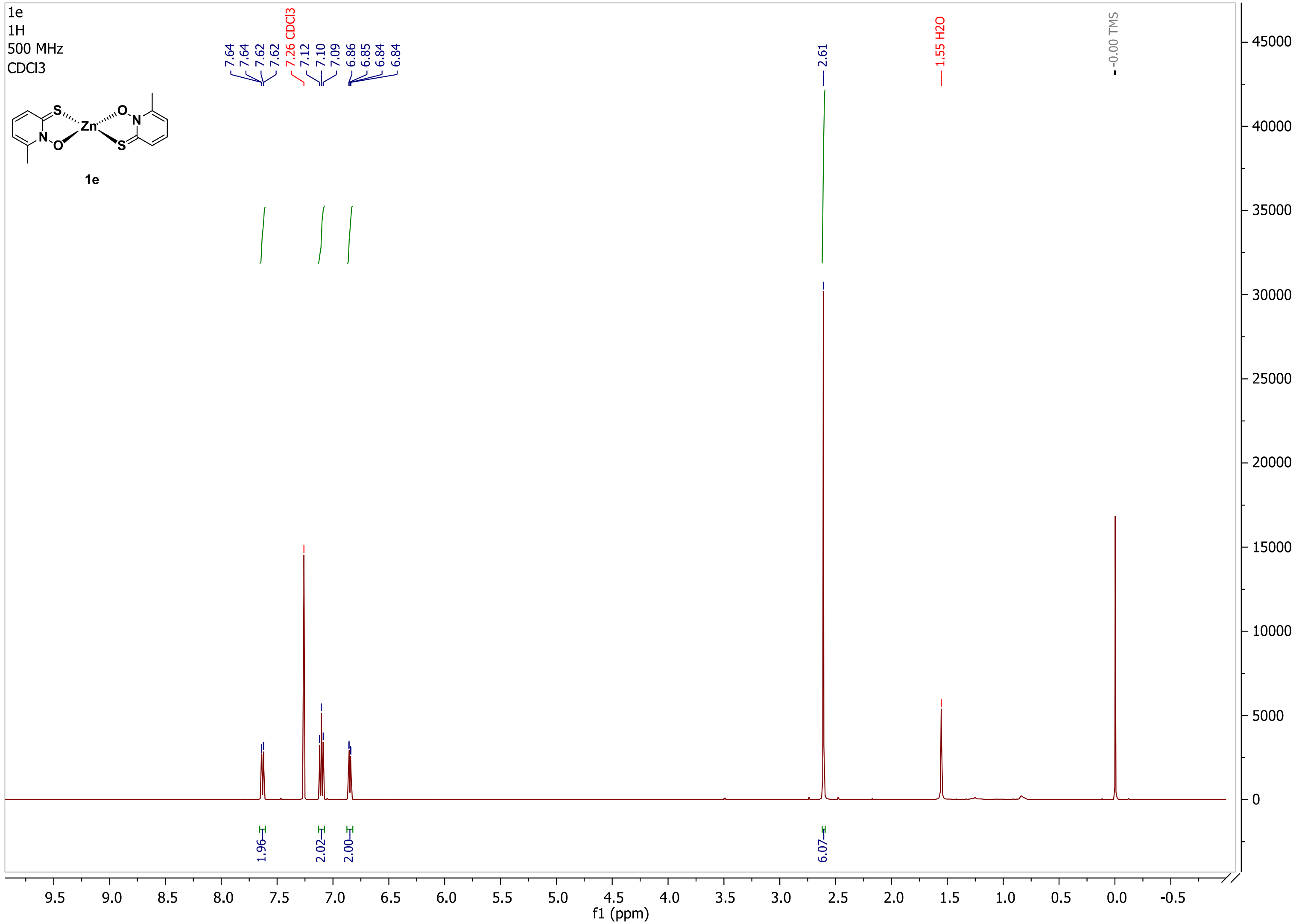

Supplementary Figure 7: ^{1}H NMR spectrum of **1e**.

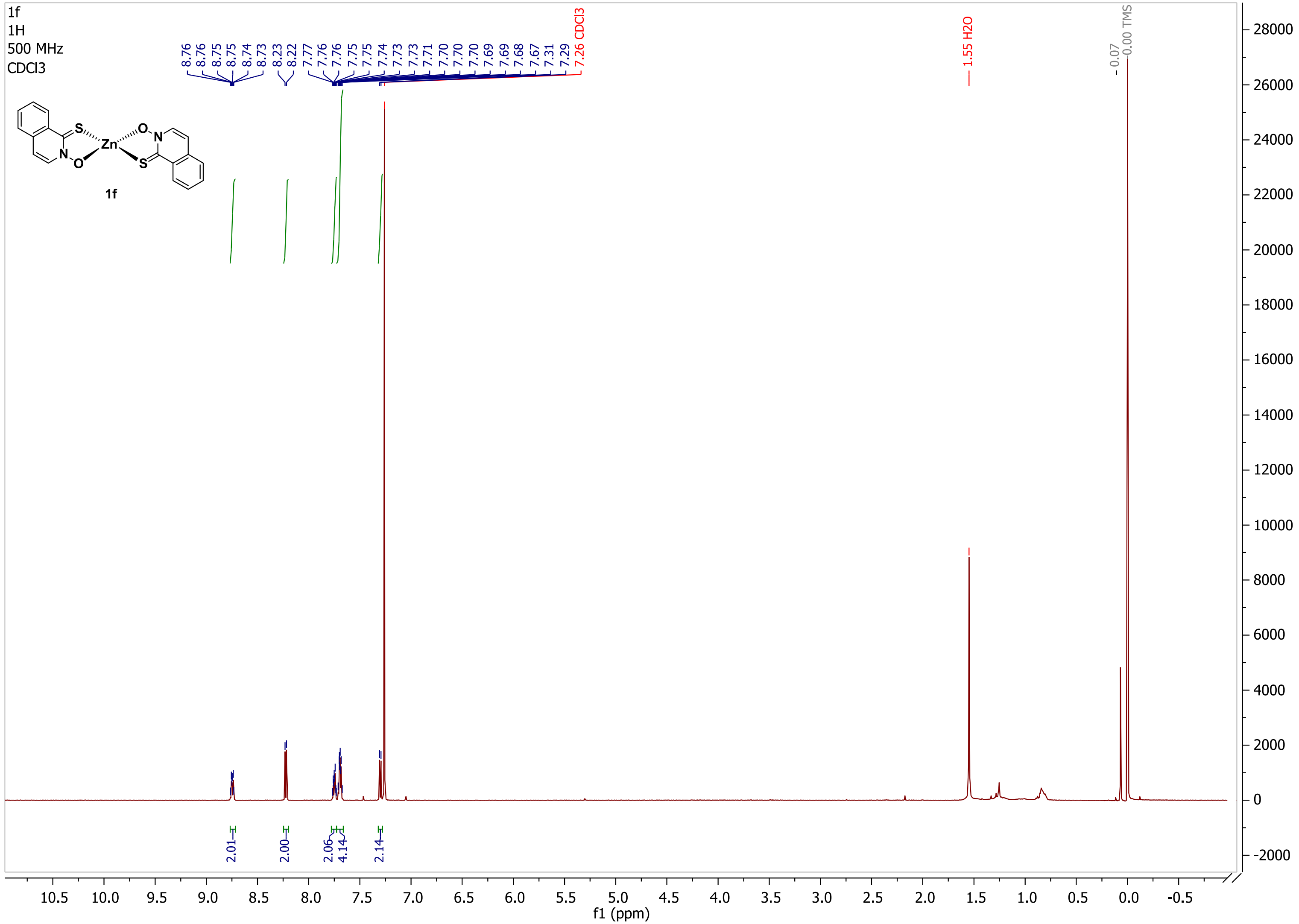

Supplementary Figure 8: ^{1}H NMR spectrum of **1f**.

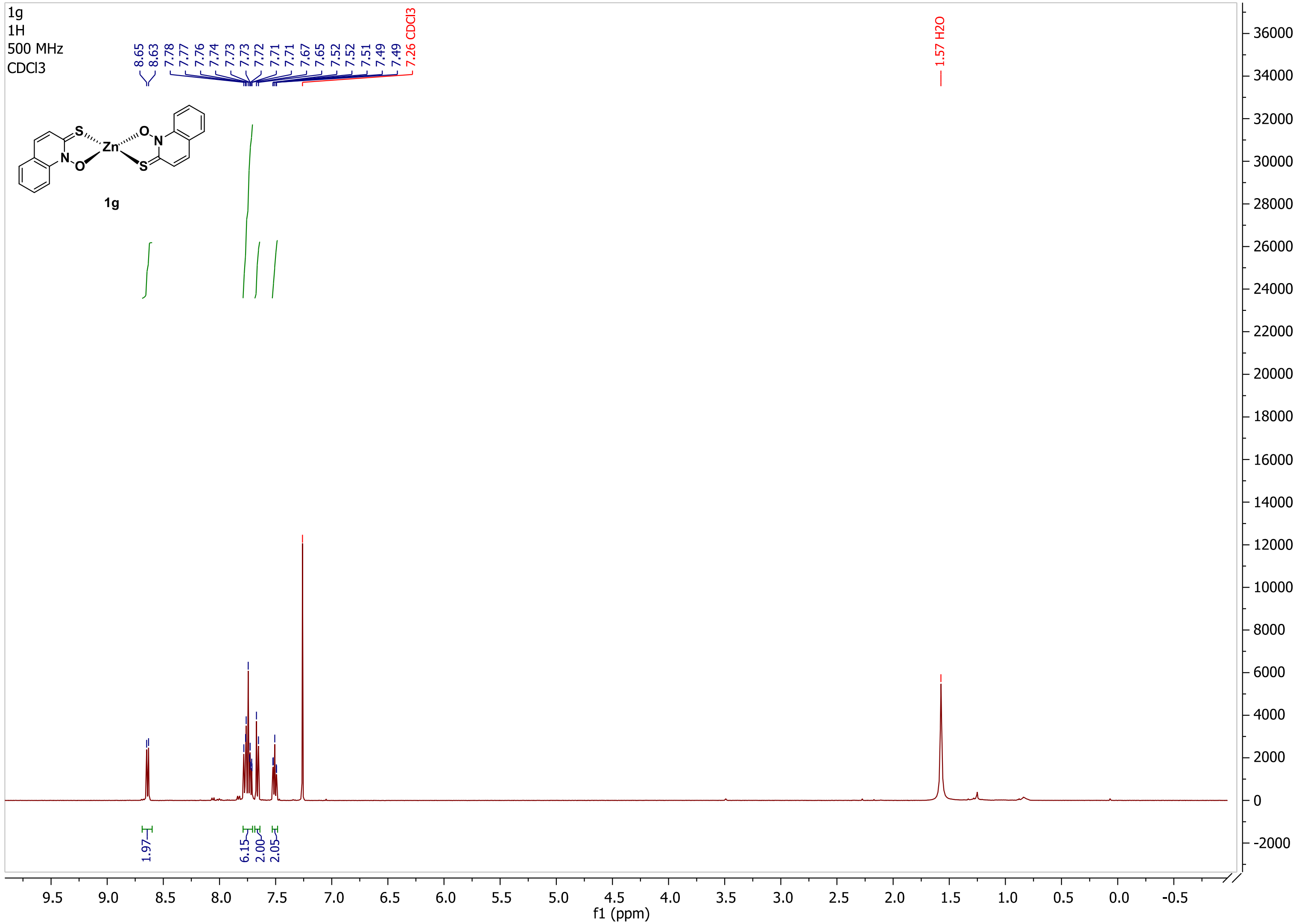

Supplementary Figure 9: ^{1}H NMR spectrum of **1g**.

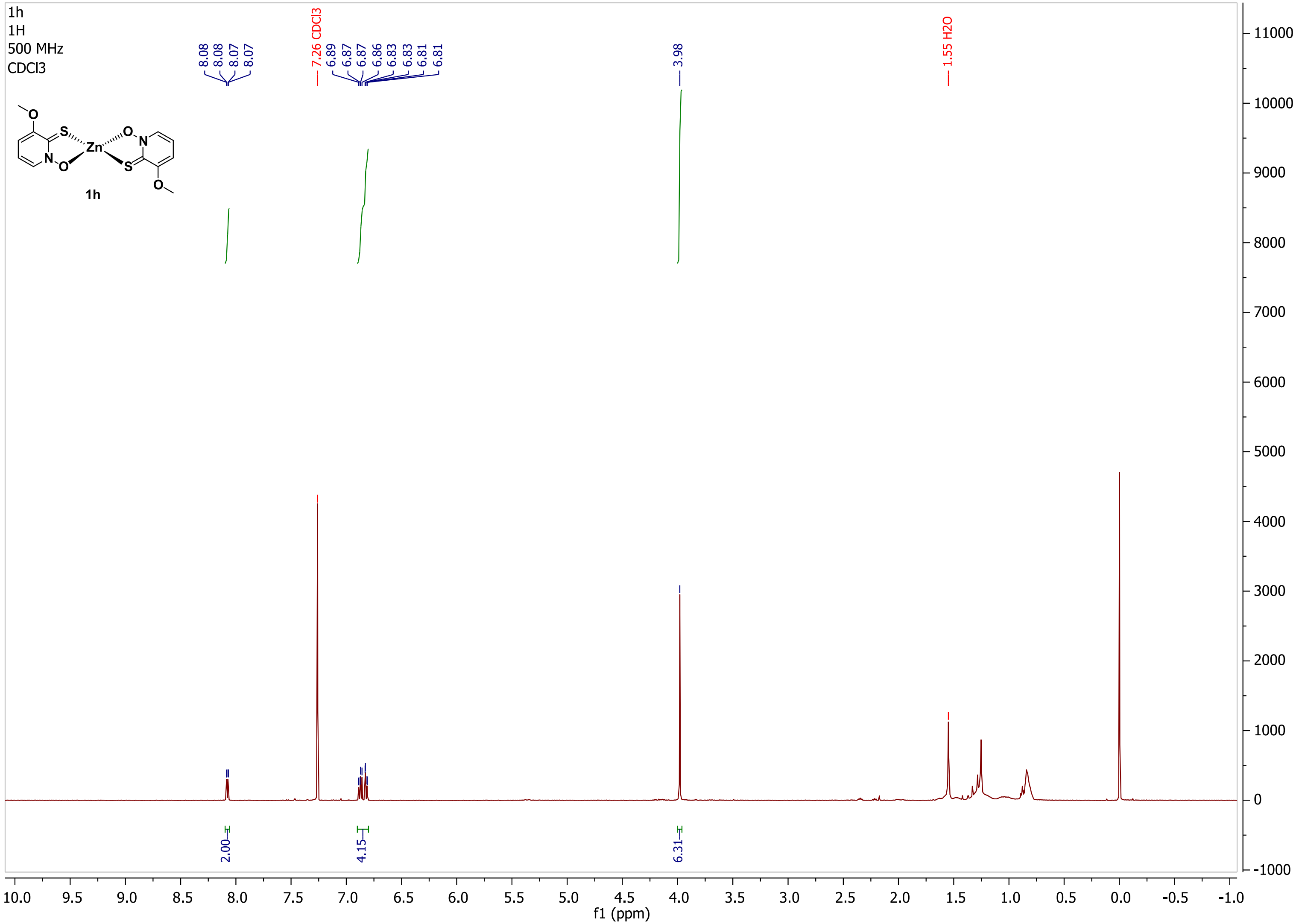

Supplementary Figure 10: ^{1}H NMR spectrum of **1h**.

5. UV-vis and NMR stability

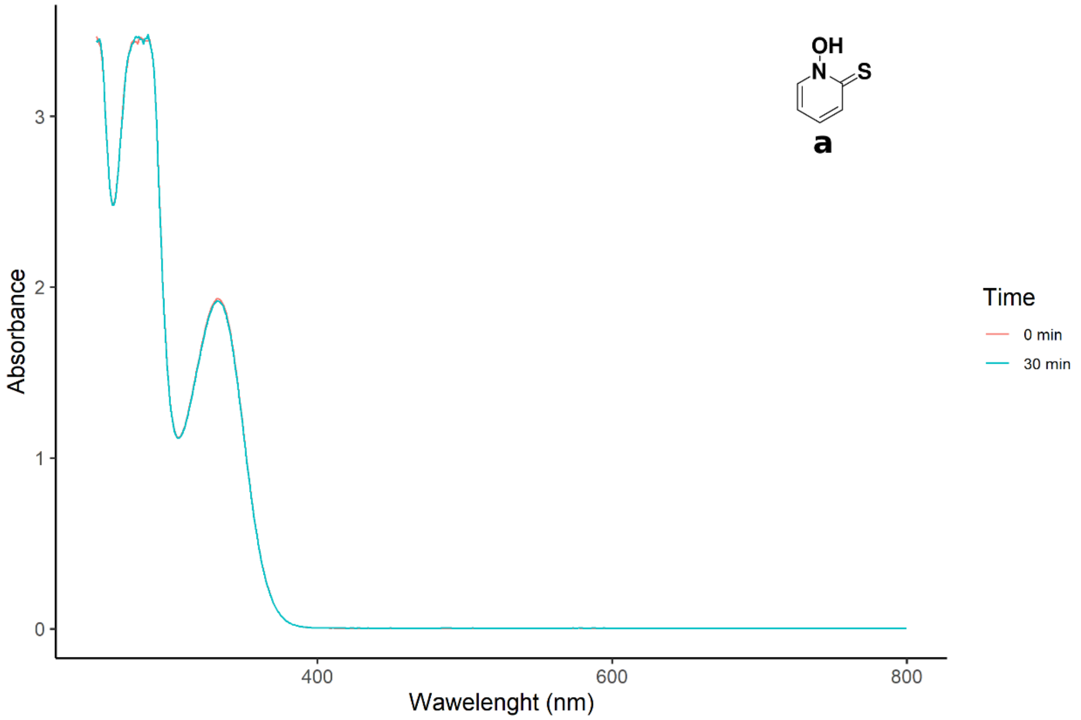

Supplementary Figure 11: UV-vis stability spectra of ligand pyrithione **a** in 1% DMSO/acetate buffer.

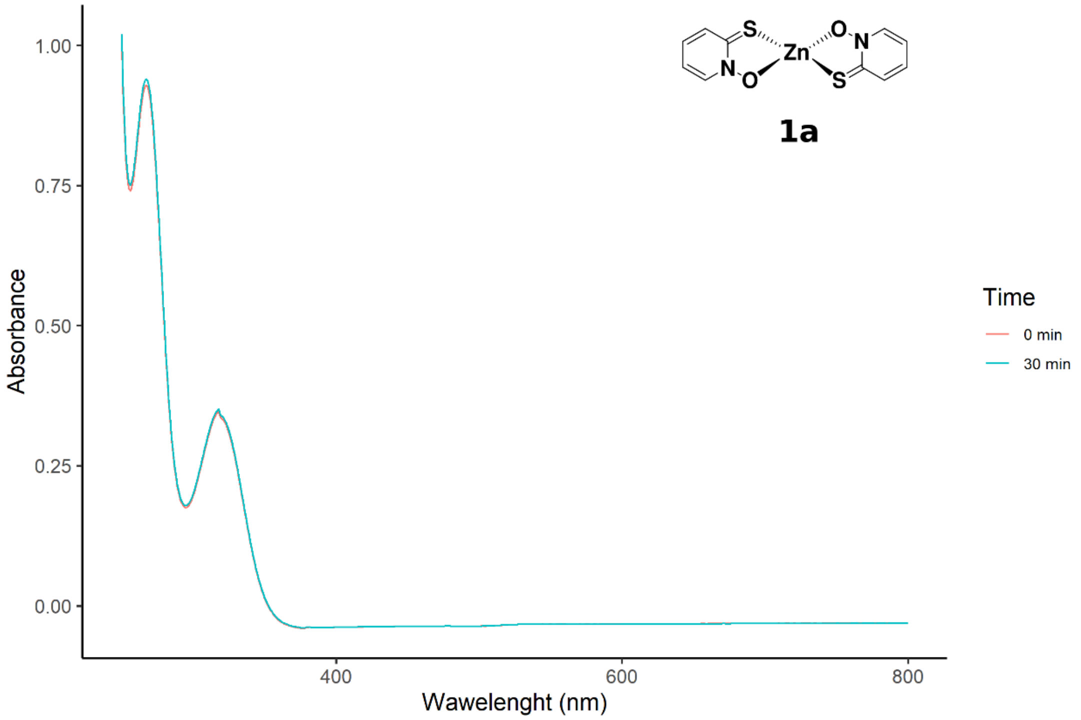

Supplementary Figure 12: UV-vis stability spectra of zinc complex **1a** in 1% DMSO/acetate buffer.

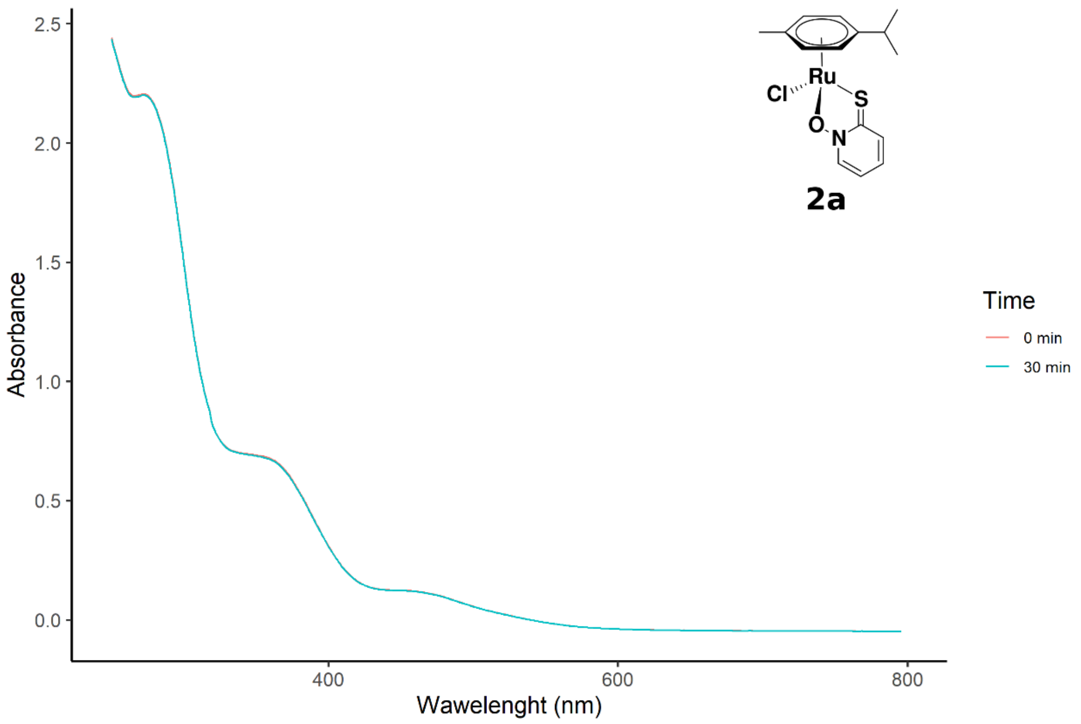

Supplementary Figure 13: UV-vis stability spectra of ruthenium complex **2a** in acetate buffer.

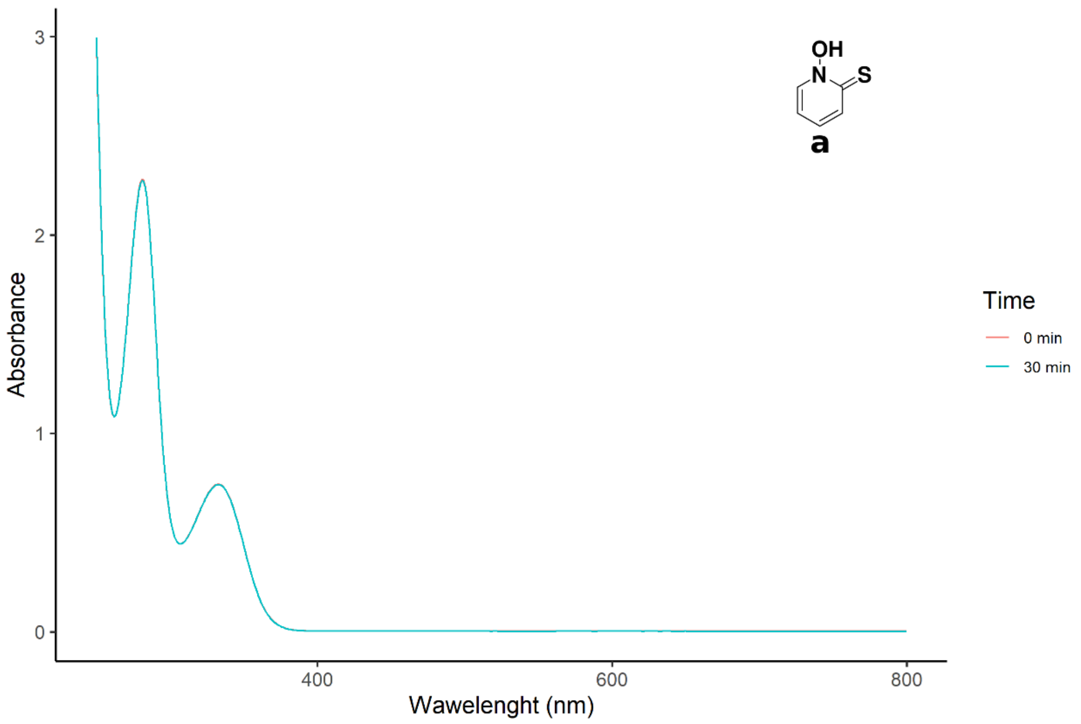

Supplementary Figure 14: UV-vis stability spectra of ligand pyrithione **a** in 1% DMSO/HEPES buffer.

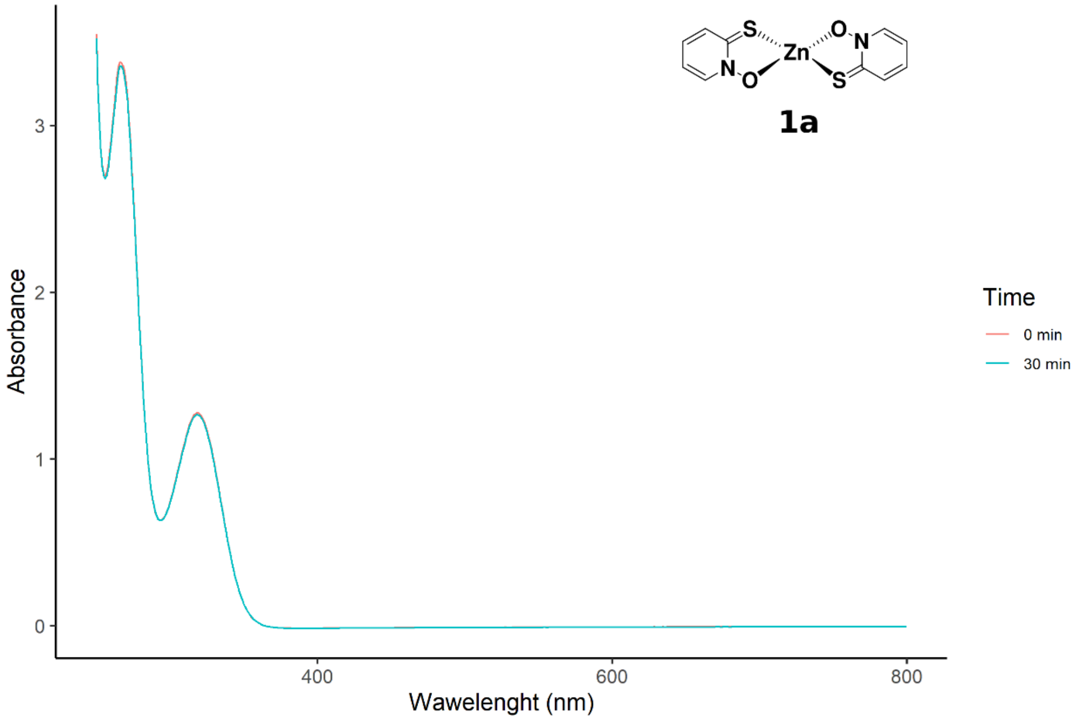

Supplementary Figure 15: UV-vis stability spectra of zinc complex **1a** in 1% DMSO/HEPES buffer.

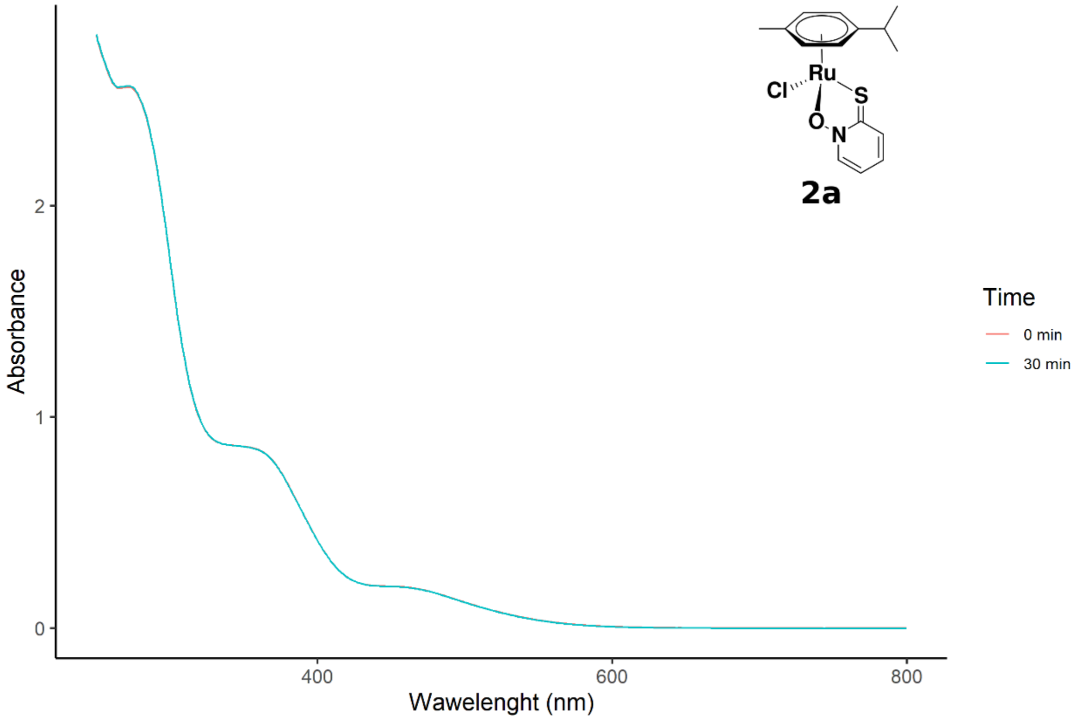

Supplementary Figure 16: UV-vis stability spectra of ruthenium complex **2a** in HEPES buffer.

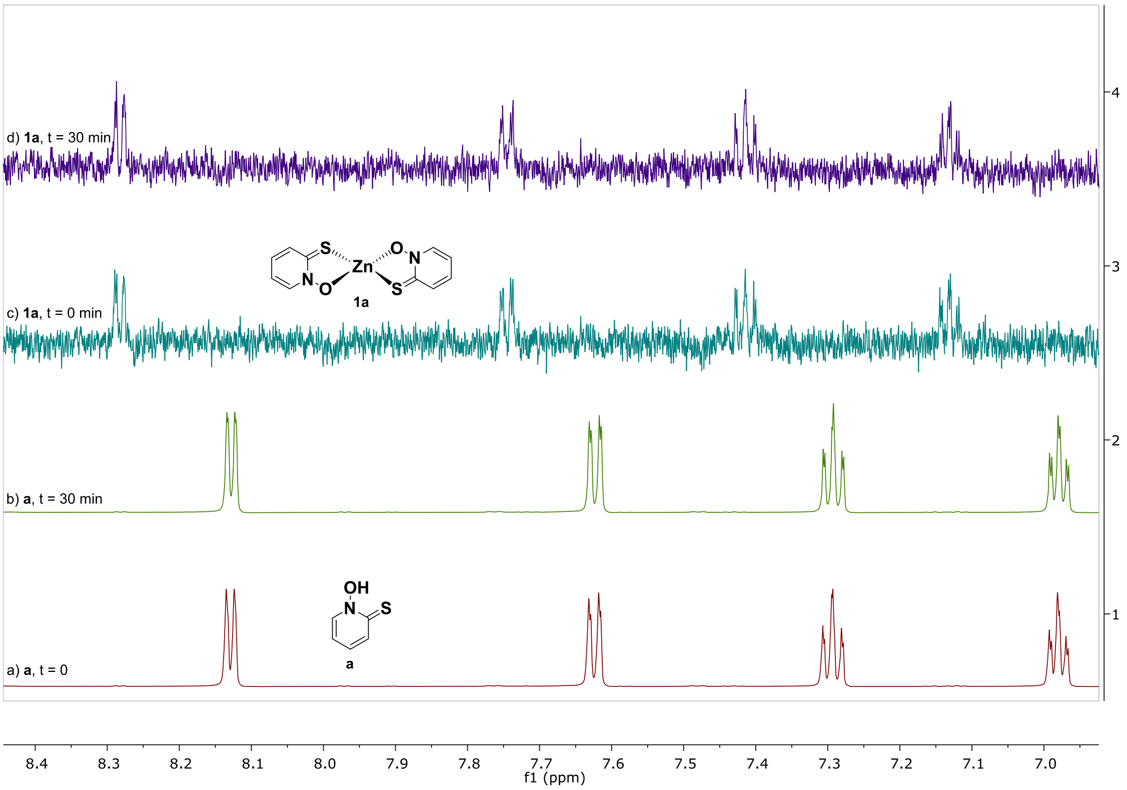

Supplementary Figure 17: ^{1}H NMR stability spectra of pyrithione **a** (a–b) and zinc complex **1a** (c–d) in 1% DMSO-d_6/acetate buffer solution prepared in D_2O recorded immediately after preparation and after 30 min.

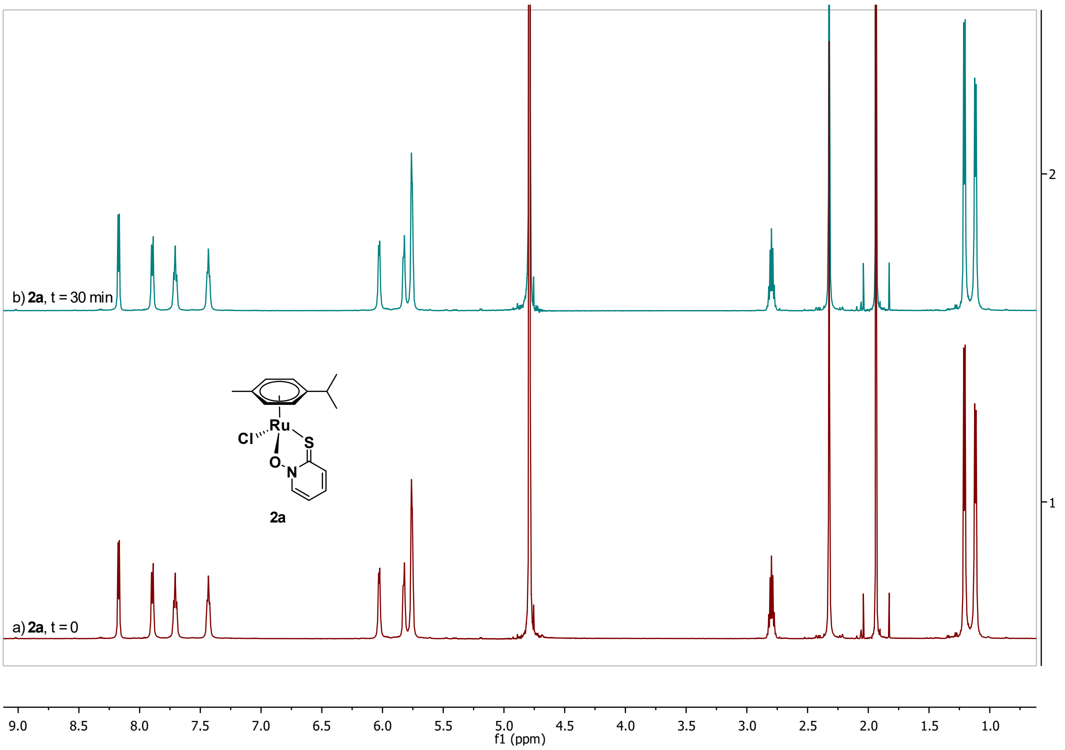

Supplementary Figure 18: ^{1}H NMR stability spectra of ruthenium complex with pyrithione **2a** in acetate buffer solution prepared in D_2O recorded immediately after preparation and after 30 min.

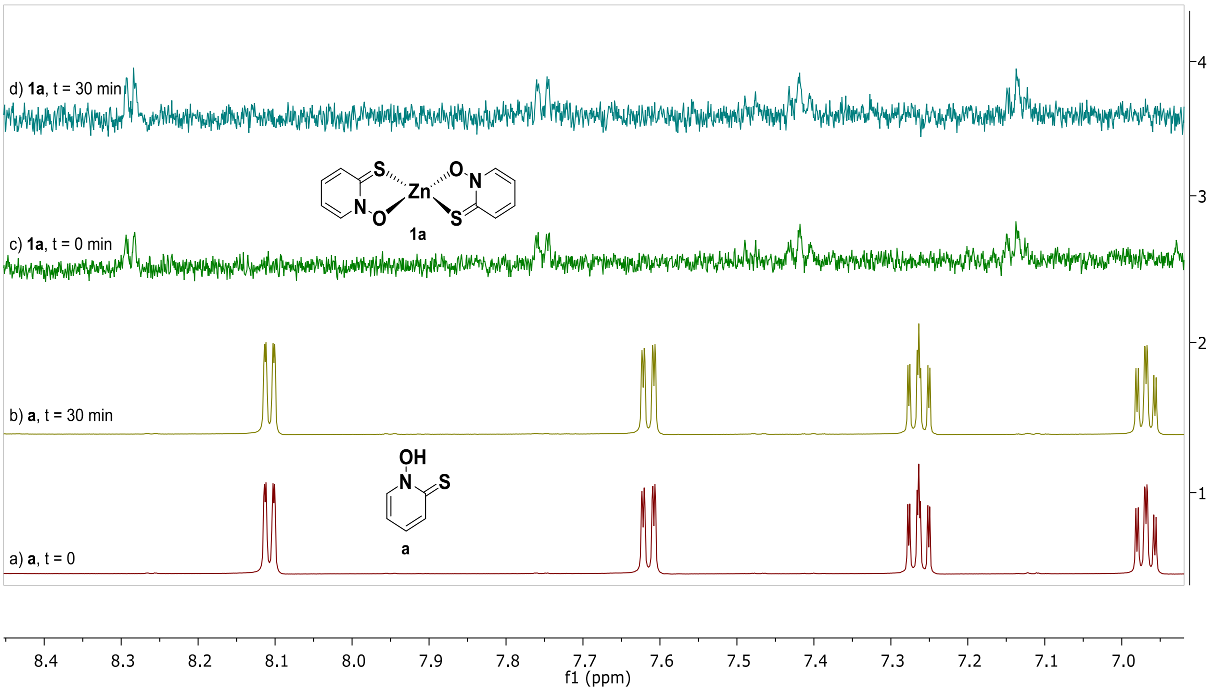

Supplementary Figure 19: ^{1}H NMR stability spectra of pyrithione **a** (a–b) and zinc complex **1a** (c–d) in 1% DMSO-d_6/HEPES buffer solution prepared in D_2O recorded immediately after preparation and after 30 min.

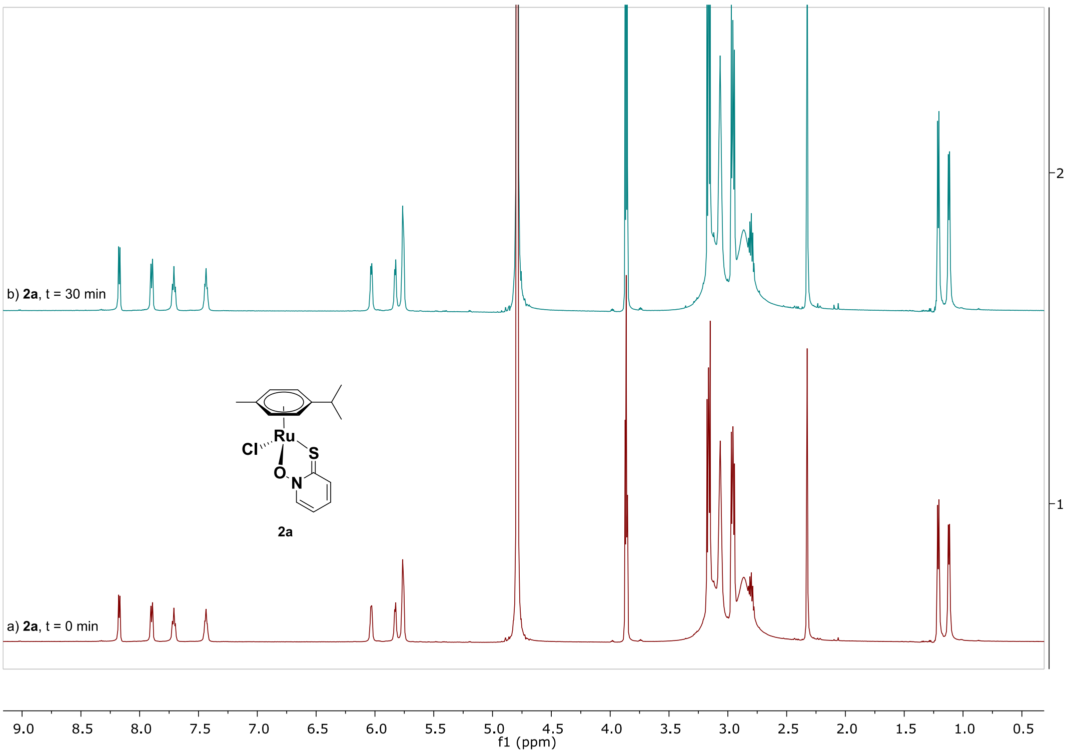

Supplementary Figure 20: ^{1}H NMR stability spectra of ruthenium complex with pyrithione **2a** in HEPES buffer solution prepared in D_2O recorded immediately after preparation and after 30 min.

6. Enzyme assays

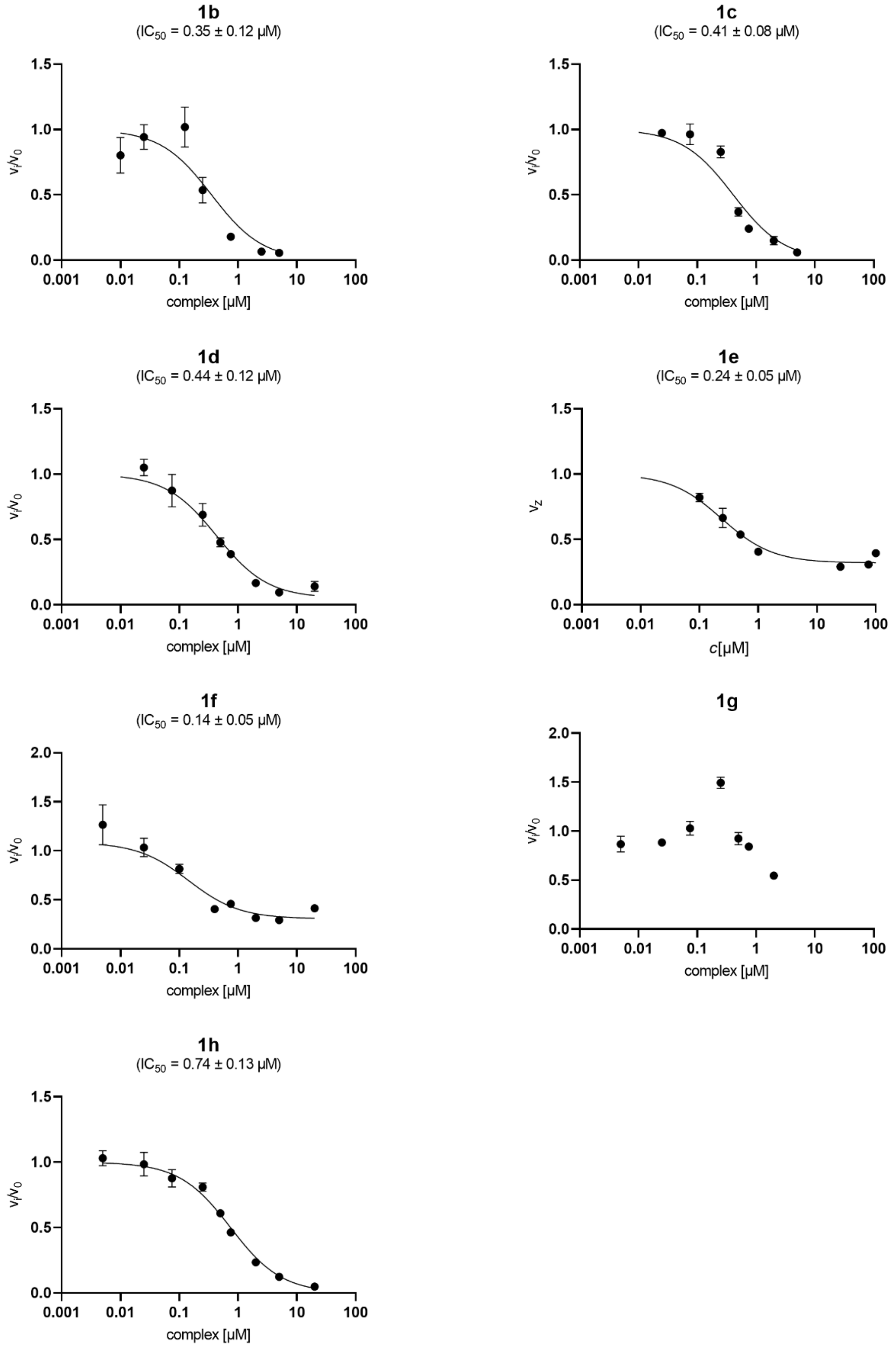

Supplementary Figure 21: Results of enzyme inhibition assays for cathepsin L. Data are mean ± s.e.m. of three measurements.

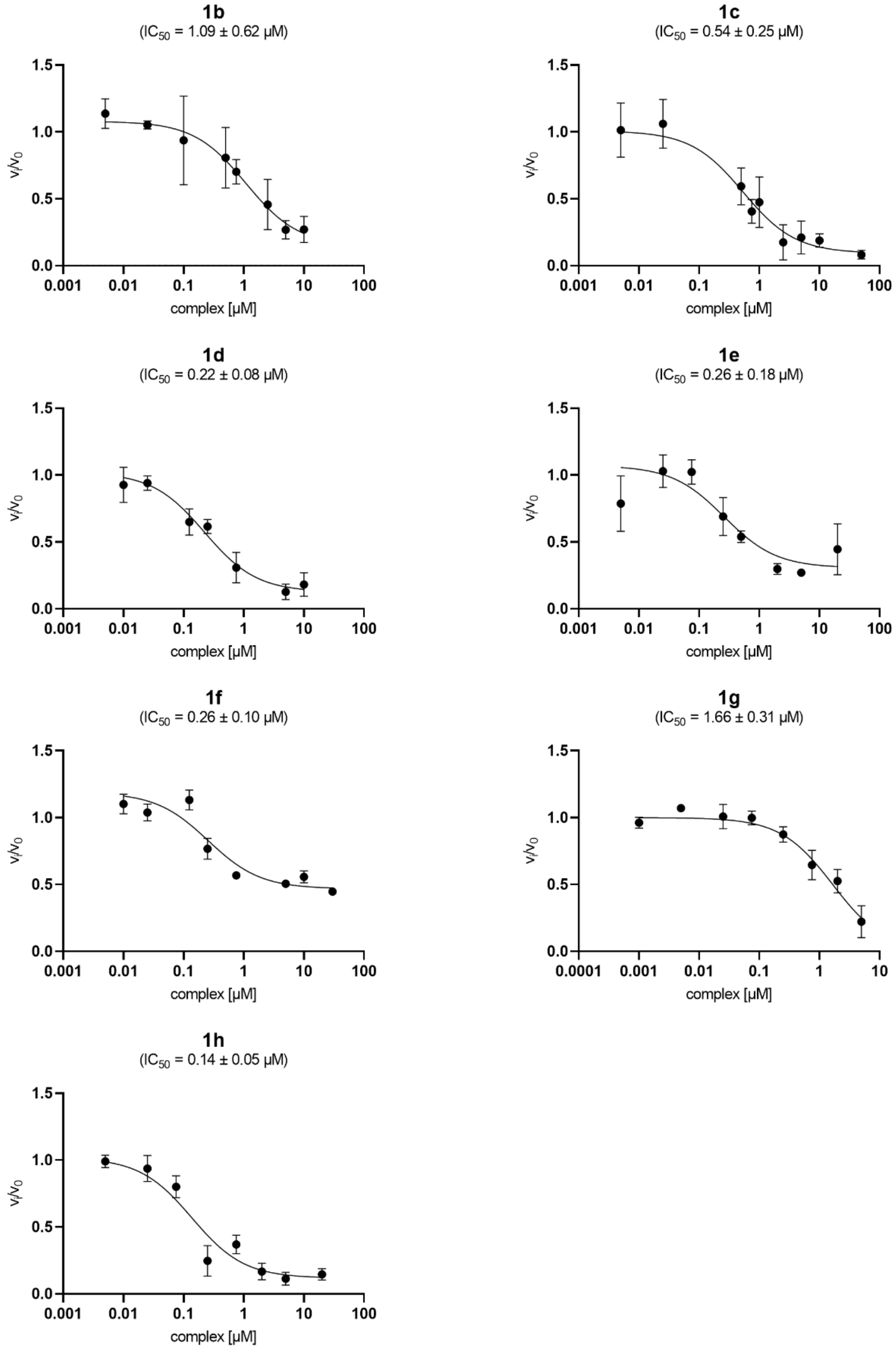

Supplementary Figure 22: Results of enzyme inhibition assays for PL^{Pro}. Data are mean ± s.e.m. of three measurements.

Supplementary Table 3: Relative reaction rates for ligands tested on Cahtepsin L and PL^{Pro}. Data are averages of three measurements.

	v_i/v_0 at 100 µM	
**Ligand**	**Cathepsin L**	**PL^{Pro}**
**a (Pth)**	1.09	0.88
**b (3MePth)**	1.05	0.50
**c (4MePth)**	1.04	0.60
**d (5MePth)**	1.01	0.77
**e (6MePth)**	1.15	0.82
**f (1iQth)**	0.65	0.66
**g (2Qth)**	0.53	0.28
**h (3OMePth)**	1.17	0.60

Supplementary Table 4: Determined IC_{50} values for zinc and ruthenium complexes towards cathepsin L and SARS-CoV-2 PL^{Pro}. Data are presented as mean ± s.e.m. (N = 3).

	IC_{50} [µM]	
**Complex**	**Cathepsin L**	**PL^{Pro}**
**1a**	1.88 ± 0.49	0.50 ± 0.07
**1b**	0.35 ± 0.12	1.09 ± 0.62
**1c**	0.41 ± 0.08	0.54 ± 0.25
**1d**	0.44 ± 0.12	0.22 ± 0.08
**1e**	0.24 ± 0.05	0.26 ± 0.18
**1f**	0.14 ± 0.05	0.26 ± 0.10
**1g**	NA	1.66 ± 0.31
**1h**	0.74 ± 0.13	0.14 ± 0.05
**2a**	116 ± 23	14.52 ± 2.49

7. Prediction data for the key parameters in drug design

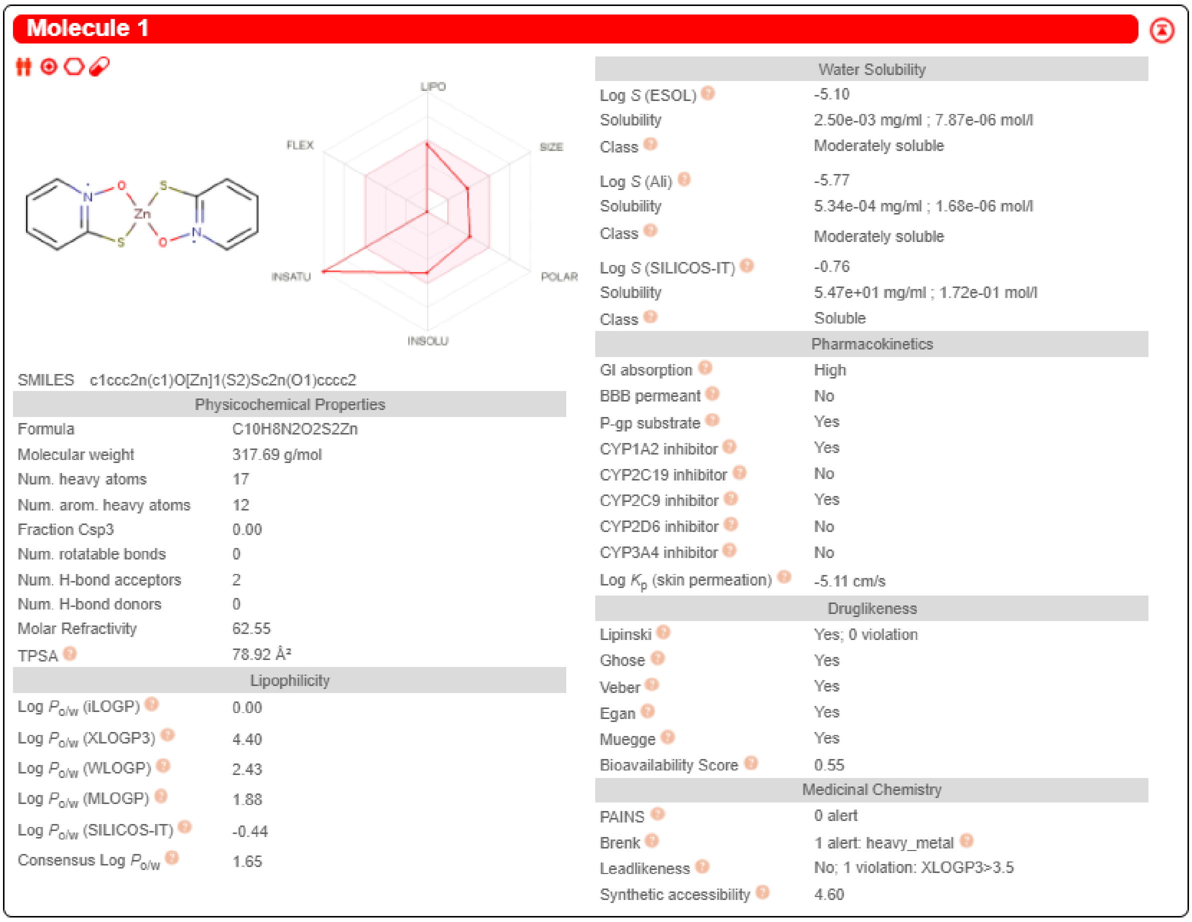

SMILES c1ccc2n(c1)O[Zn]1(S2)Sc2n(O1)cccc2

Physicochemical Properties	
Formula	C10H8N2O2S2Zn
Molecular weight	317.69 g/mol
Num. heavy atoms	17
Num. arom. heavy atoms	12
Fraction Csp3	0.00
Num. rotatable bonds	0
Num. H-bond acceptors	2
Num. H-bond donors	0
Molar Refractivity	62.55
TPSA	78.92 Å²

Lipophilicity	
Log $P_{o/w}$ (iLOGP)	0.00
Log $P_{o/w}$ (XLOGP3)	4.40
Log $P_{o/w}$ (WLOGP)	2.43
Log $P_{o/w}$ (MLOGP)	1.88
Log $P_{o/w}$ (SILICOS-IT)	-0.44
Consensus Log $P_{o/w}$	1.65

Water Solubility	
Log S (ESOL)	-5.10
Solubility	2.50e-03 mg/ml ; 7.87e-06 mol/l
Class	Moderately soluble
Log S (Ali)	-5.77
Solubility	5.34e-04 mg/ml ; 1.68e-06 mol/l
Class	Moderately soluble
Log S (SILICOS-IT)	-0.76
Solubility	5.47e+01 mg/ml ; 1.72e-01 mol/l
Class	Soluble

Pharmacokinetics	
GI absorption	High
BBB permeant	No
P-gp substrate	Yes
CYP1A2 inhibitor	Yes
CYP2C19 inhibitor	No
CYP2C9 inhibitor	Yes
CYP2D6 inhibitor	No
CYP3A4 inhibitor	No
Log K_p (skin permeation)	-5.11 cm/s

Druglikeness	
Lipinski	Yes; 0 violation
Ghose	Yes
Veber	Yes
Egan	Yes
Muegge	Yes
Bioavailability Score	0.55

Medicinal Chemistry	
PAINS	0 alert
Brenk	1 alert: heavy_metal
Leadlikeness	No; 1 violation: XLOGP3>3.5
Synthetic accessibility	4.60

Supplementary Figure 23: Evaluation of physicochemical properties, pharmacokinetics, drug-likeness and medicinal chemistry friendliness of zinc pyrithione **1a**.[16]

8. References

1. CrysAlis PRO; Oxford Diffraction Ltd.: Yarnton, Oxfordshire, UK, 2011.

2. Dolomanov O V, Bourhis LJ, Gildea RJ, Howard JAK, Puschmann H. OLEX2: a complete structure solution, refinement and analysis program. J Appl Crystallogr 2009;42:339–341.

3. Sheldrick, G.M. SHELXL2018/3; University of Göttingen: Göttingen, Germany, 2018.

4. Macrae CF, Edgington PR, McCabe P, Pidcock E, Shields GP, Taylor R, Towler M, van De Streek J. Mercury: visualization and analysis of crystal structures. J Appl Cryst 2006;39:453–457.

5. CCDC. Available from: https://www.ccdc.cam.ac.uk/structures/.

6. Martin DP, Blachly PG, McCammon JA, Cohen SM. Exploring the influence of the protein environment on metal-binding pharmacophores. J Med Chem 2014;57:7126–7135.

7. Kladnik J, Kljun J, Burmeister H, Ott I, Romero-Canelón I, Turel I. Towards identification of essential structural elements of organoruthenium(II)-pyrithionato complexes for anticancer activity. Chem Eur J 2019;25:14169–14182.

8. Kladnik J, Ristovski S, Kljun J, Defant A, Mancini I, Sepčić K, Turel I. Structural isomerism and enhanced lipophilicity of pyrithione ligands of organoruthenium(II) complexes increase inhibition on AChE and BuChE. Int J Mol Sci 2020;21:5628.

9. Magda D, Lecane P, Wang Z, Weilin H, Thiemann P, Ma X, Dranchak PK, Wang X, Lynch V, Wei W, et al. Synthesis and anticancer properties of water-soluble zinc ionophores. Cancer Res. 2008;68:5318–5325.

10. Barnett BL, Kretschmar HC, Hartman FA. Structural characterization of bis(*N*-oxopyridine-2-thionato)zinc(II). Inorg Chem 1977;16:1834–1838.

11. Xiong RG, Song BL, You XZ, Mak TCW, Zhou ZY. Syntheses and properties of some transition metal complexes with methyl substituted 1-hydroxy-2(1*H*)-pyridinethione and crystal structure of bis(1-hydroxy-4-methyl-2(1*H*)pyridinethionato-*O,S'*)zinc(II). Polyhedron. 1996;15:991–996.

12. West DX, Brown CA, Jasinski JP, Jasinski JM, Heathwaite RM, Fortier DG, Staples RJ, Butcher RJ. Crystal structures of the cobalt(III), nickel(II), copper(II), and zinc(II) complexes of 2-thio-6-picoline *N*-oxide. J Chem Crystallogr 1998;28:853–860.

13. Yang L, Powell DR, Houser RP. Structural variation in copper(I) complexes with pyridylmethylamide ligands: structural analysis with a new four-coordinate geometry index, τ4. Dalton Trans 2007;955–964.

14. Okuniewski A, Rosiak D, Chojnacki J, Becker B. Coordination polymers and molecular structures among complexes of mercury(II) halides with selected 1-benzoylthioureas. Polyhedron. 2015;90:47–57.

15. Addison AW, Rao TN, Reedijk J, Vanrijn J, Verschoor GC. Synthesis, structure, and spectroscopic properties of copper(II) compounds containing nitrogen–sulphur donor ligands; the crystal and molecular structure of aqua[1,7-bis(*N*-methylbenzimidazol-2′-yl)-2,6-dithiaheptane]copper(II) perchlorate. J Chem Soc Dalton Trans 1984;1349–1356.

16. SwissADME. [cited 2022 Jan 25]. Available from: http://www.swissadme.ch/index.php.
